# Sensitivity to NH_3_ Vapor: Synthesis and Characterization of Five New Coordination Polymers Based on 2,2-Dimethylglutaric Acid and Bis(triazole)-Derived Ligands

**DOI:** 10.1021/acsomega.3c03212

**Published:** 2024-01-08

**Authors:** Pelin Köse Yaman, Özge Demir, Sevde Demir, Merve Zeyrek Ongun, Sibel Oğuzlar, Hakan Erer

**Affiliations:** † Department of Chemistry, Faculty of Science, Dokuz Eylül University, 35390 İzmir, Türkiye; ‡ Department of Chemistry, Faculty of Science, Eskişehir Osmangazi University, 26040 Eskişehir, Türkiye; § Chemistry Technology Program, İzmir Vocational High School, Dokuz Eylül University, 35210 İzmir, Türkiye; ∥ Center for Fabrication and Application of Electronic Materials, Dokuz Eylül University, 35390 İzmir, Türkiye

## Abstract

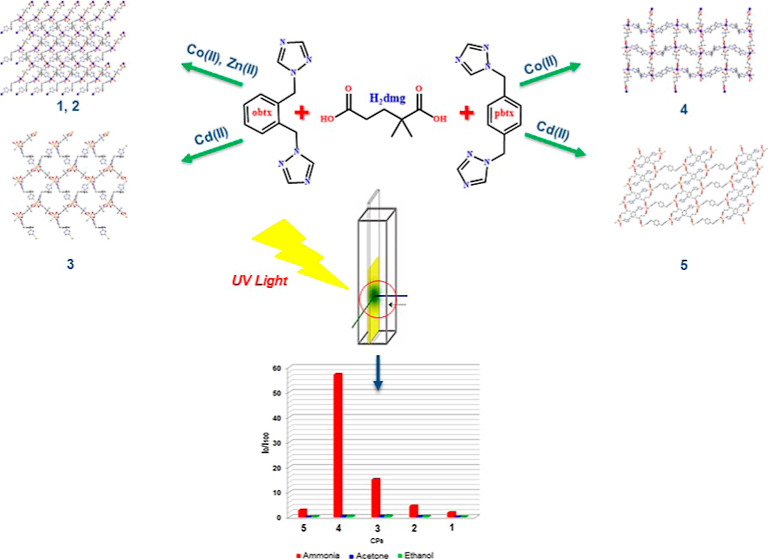

Five new coordination polymers (CPs) were obtained as a result of hydrothermal reactions of 2,2-dimethylglutaric acid (H_2_dmg) and 1,4-bis(1*H*-1,2,4-triazol-1-ylmethyl)benzene (**pbtx**)/1,2-bis(1*H*-1,2,4-triazol-1-ylmethyl)benzene (**obtx**) ligands with some metal ions [Co(μ-dmg)(μ-obtx)]_*n*_ (**1**), [Zn(μ-dmg)(μ-obtx)]_*n*_ (**2**), [Cd(μ-dmg)(μ-obtx)]_*n*_ (**3**), [Co_2_(μ-dmg)_2_(μ-pbtx)_2_]_*n*_ (**4**), and [Cd(μ-dmg)(H_2_O)(μ-pbtx)]_*n*_ (**5**). All of the compounds were characterized by elemental analysis, FT-IR spectroscopy, single-crystal X-ray diffraction, powder X-ray diffraction, and thermal analysis techniques. The single-crystal X-ray studies show that all compounds exhibit 2D layer structures. To examine the ammonia sensing properties of five new coordination complexes (**1–5**), the absorption and emission spectra of CPs embedded in ethyl cellulose thin films were measured by exposure to different concentrations of ammonia (NH_3_) vapor. The [Co_2_(μ-dmg)_2_(μ-pbtx)_2_]_*n*_ (**4**)-based sensor agent was found to show promising sensor properties in detecting NH_3_ vapor.

## 1 Introduction

Coordination polymers (CPs) were created by linking together many themes of carboxylic acids, metal salts, and occasionally supplementary organic ligands.^1−3^ For prospective uses such as sensors, catalysts, medication delivery, and gas storage, CPs have recently advanced.^2−7^ The variety of architectures and topologies of CPs are also of great interest. However, choosing optimal donor atom ligands as well as reaction circumstances like the right solvent system pose significant challenges to the intelligent and specialized design of CPs.^8^ In general, organic linkers with a specific symmetry are crucial for creating CPs with the appropriate characteristics. Bis(triazole) compounds have garnered a lot of attention as linkers at this time.^9−11^

As such, secondary (neutral) ligands, symmetrical structures containing two donor atoms and aromatic rings, are generally preferred. To obtain more porous and durable CPs, at least a ligand used should have unbending or semiflexible properties.^12^ Recently, secondary (neutral) ligands have been used to fill the coordination gap of the metal and increase the dimensionality. With the use of neutral ligands, CPs can be synthesized more easily and the structural diversity and their dimensionality can be increased.^13^ It is very practical to synthesize CPs with functional properties arising from geometry by determining suitable metal ions and ligands. Also, the combination of dicarboxylate and N-donor ligands may provide opportunities for structural diversity in self-assembly.^14^

Major environmental problems include the negative effects on air quality caused by increased emissions of volatile organic compounds (VOCs).^15,16^ Many analytical techniques such as mass spectrometry, chromatography, nuclear magnetic resonance, etc. have been used for accurate quantification of VOCs, but such techniques have disadvantages such as portability and high cost. As this is the case, various sensing principles and materials have been used to examine more cost-effective methods, some of which are conductive polymers, quartz crystal microbalance sensors, semiconductor metal oxides, etc. However, such sensors often suffer from limitations in selectivity, interference (from moisture), sensitivity, reproducibility, stability, or false response due to sensor aging.^17−19^

The VOC emissions into the environment are not desired since they will have a negative impact on both human health and environmental quality. Coordination polymer/metal–organic frameworks (CP/MOFs) have garnered significant attention in the creation of next-generation sensing devices and have provided solutions to these issues. Because of their porosity, CPs work well as adsorbents for gaseous molecules, solvent vapors, and VOCs, among other things.^20^ Recently, investigations have indicated that CPs have a potentially significant role in the monitoring and analysis of volatile molecules.^21^ Zou et al. carried out a study using MOFs in thin film forms for the determination of VOCs in water, ethanol, and acetonitrile solutions and showed that the highly selective and sensitive detection of dimethylamine is by fluorescent quenching mechanism.^21,22^ Some MOFs were doped with lanthanide group elements (Eu, Tb) to provide open positions, and their effects on different solvents were investigated.^23,24^ While we carried out our studies at room temperature, some studies in the literature were realized at 100 °C.^25,26^ The ammonia sensor experiments of Peng et al. and Wong et al. experiments in the UV region draw attention.^27,28^ Zhang et al. reported that ultrasensitive room-temperature NH_3_ sensors were prepared by assembling carbon quantum dots on free-standing ultrathin CP nanosheets.^29^ AgBr and the ligand TabHPF_6_ were combined by Wang and colleagues to produce the one-dimensional CP [(TabH)(AgBr_2_)]_*n*_, which contains hydrogen bonds between the cationic thiols TabH^+^ and the anionic chains [AgBr_2_]_*n*_^*n*–^. They reported that the sensor response *R*_a_/*R*_0_ was as high as 197 when the ammonia concentration was 3000 ppm. Below 1000 ppm, good linearity between the sensitivity and concentration was seen. The response of *R*_a_/*R*_0_ was 2.5 when the concentration of NH_3_ in water at room temperature dropped to 30 ppm.^30^ Stevens et al. described a unique technique that includes trapping and gluing the apochromatic CPs to the sensor surface using a sheet of post arrays made of polydimethylsiloxane. They claimed that when exposed to 1000 ppm of ammonia, optical tests revealed that the spectral peaks had widened and the reaction time had slowed in comparison to responses from greater ammonia concentrations.^31^

In this study, two flexible bis(triazole) derivative ligands, namely, 1,4-bis((1*H*-1,2,4-triazol-1-yl)methyl)benzene (**pbtx**) and 1,2-bis((1*H*-1,2,4-triazol-1-yl)methyl)benzene (**obtx**) were synthesized and their five Co(II), Zn(II), and Cd(II) compounds, [Co(μ-dmg)(μ-obtx)]_*n*_ (**1**), [Zn(μ-dmg)(μ-obtx)]_*n*_ (**2**), [Cd(μ-dmg)(μ-obtx)]_*n*_ (**3**), [Co_2_(μ-dmg)_2_(μ-pbtx)_2_]_*n*_ (**4**), and [Cd(μ-dmg)(H_2_O)(μ-pbtx)]_*n*_ (**5**), were obtained with 2,2-dimethylglutaric acid (H_2_dmg). Elemental analysis, FT-IR spectroscopy, and single-crystal X-ray diffraction (SCXRD) methods were used to explain and elucidate the obtained compounds. Simultaneously, the thermal properties of the compounds were investigated. The performance tests of the synthesized 2D compounds against ammonia (NH_3_) were carried out based on the emission intensity measurement in the form of ethyl cellulose (EC) thin films with fluorescence lifetime and a steady-state spectrometer.

## 2 Materials and Methods

### 2.1 Materials

The obtx and pbtx ligands were synthesized according to the literature^32^ [the structures of obtx and pbtx ligands are elucidated by ^1^H NMR spectra (Figures S1 and S2)]. Solvents for the spectroscopic studies, metal salts, and other chemicals were used without further purification. 2,2-dimethylglutaric acid (Merck) and all of the metal salts, Co(NO_3_)_2_·6H_2_O, Zn(NO_3_)_2_·6H_2_O, Cd(OAc)_2_·2H_2_O, and Cd(SO_4_)_2_·2/3H_2_O (Sigma-Aldrich), were commercially purchased. Tetrahydrofuran (THF) and ammonia (NH_3_) were obtained from Fluka. The polymer, EC, and plasticizer, dioctyl phthalate (DOP), were from Sigma-Aldrich.

### 2.2 Instruments

^1^H NMR spectra of obtx and pbtx ligands were captured on a 500.13 MHz JEOL ECZ 500R instrument at room temperature. The resulting compounds **1–5** were characterized by elemental analyses, FT-IR spectra, SCXRD, powder X-ray diffraction (PXRD), and thermal analysis (TG/DTA) techniques. Elemental analysis results are consistent with single-crystal X-ray results. Elemental analyses (C, H, and N) were performed on a (C, H, N) LECO, CHNS-932. FT-IR spectra of the compounds were carried out at room temperature by a PerkinElmer FT-IR 100 spectrometer in the region of 4000–400 cm^–1^. PXRD patterns were collected by a Panalytical Empyrean X-ray diffractometer in the 2θ range of 55 5–50° Cu Kα radiation (λ = 1.5406 Å). The thermograms were collected via thermogravimetric analysis (TGA) measurements using a PerkinElmer Diamond TG/DTA thermal analyzer in a static air atmosphere at a heating rate of 10 °C min^–1^ in the temperature range of 30–700 °C. The emission and excitation spectra of the compounds were measured by an Edinburgh FLSP920 spectrometer operating on the time-dependent single-photon counting (TCSPC) principle. Suitable crystals of **1–5** were selected for data collection, which was performed on a Bruker APEX-II diffractometer equipped with a graphite-monochromatic Mo–Kα radiation at 293 K. The structures were solved by intrinsic phasing methods using the program SHELXT-2015 in OLEX2^33^ with anisotropic thermal parameters for all non-hydrogen atoms. All non-hydrogen atoms were refined anisotropically by full-matrix least-squares methods using SHELXL-2015,^34^ and structural figures were obtained using Mercury.^35^ Crystal data and structure refinement parameters for the compounds are presented in Table 1. Selected bond distances and angles of compounds are given in Tables S1–S5.

**Table 1:** Crystal Data and Structure Refinement Parameters for Compounds **1–5**

	**1**	**2**	**3**	**4**	**5**
empirical formula	C_19_H_22_N_6_O_4_Co	C_19_H_22_N_6_O_4_Zn	C_19_H_22_N_6_O_4_Cd	C_38_H_44_N_12_O_8_Co_2_	C_19_H_24_N_6_O_5_Cd
formula weight	457.35	463.79	510.82	914.71	528.84
crystal system	monoclinic	monoclinic	monoclinic	orthorhombic	triclinic
space group	*P*2_1_/*n*	*P*2_1_/*n*	*P*2_1_/*n*	*Pca*2_1_	*P*1̅
*a* (Å)	6.8937(7)	6.9181(18)	7.0035(4)	17.2066(5)	9.0473(14)
*b* (Å)	13.7717(15)	13.801(3)	14.2654(9)	19.0385(5)	10.4823(16)
*c* (Å)	21.135(2)	21.148(6)	20.9182(13)	12.6514(3)	12.3814(17)
α (deg)	90.00	90.00	90.00	90.00	90.390(4)
β (deg)	95.491(3)	95.049(8)	92.067(2)	90.00	110.529(4)
γ (deg)	90.00	90.00	90.00	90.00	99.678(4)
*V* (Å3)	1997.3(4)	2011.4(9)	2088.5(2)	4144.45(19)	1081.2(3)
*Z*	4	4	4	4	2
*D*_c_ (g cm^–^^3^)	1.521	1.532	1.625	1.466	1.624
μ (mm–1)	0.90	1.26	1.08	0.87	1.05
θ range (deg)	2.4–27.9	2.4–23.0	2.4–27.3	2.6–26.4	2.9–28.4
measured refls.	50,745	41,724	45,981	66,719	57,647
independent refls.	4973	5012	5172	10,151	5353
*R*_int_	0.038	0.069	0.039	0.069	0.031
*S*	1.08	1.03	1.05	1.01	1.28
*R*1/*wR*2	0.048/0.146	0.058/0.180	0.031/0.071	0.050/0.103	0.027/0.063
⊗⟩_max_/⊗⟩_min_ (eÅ^–3^)	1.38/–0.73	1.00/–0.71	0.75/–0.52	0.65/–0.37	1.56/–0.64

### 2.3 Syntheses of Compounds **1–5**

#### 2.3.1 Synthesis of [Co(μ-dmg)(μ-obtx)]_*n*_ (**1**)

Co(NO_3_)_2_·6H_2_O (58 mg, 0.2 mmol) and 2,2-dimethylglutaric acid (32 mg, 0.2 mmol) were dissolved in distilled water (3 mL). obtx (47 mg, 0.2 mmol) was added to the previous solution and stirred for 30 min; then the mixture was sealed in a 10 mL vial and heated at 100 °C for 3 days and then cooled to room temperature at a rate of 10 °C/h. Purple block crystals of **1** in ca. 52% (based on Co) were washed with water and methanol and then dried in air. Anal. Calcd for C_19_H_22_N_6_O_4_Co (457.35 g mol^–1^) (%): C, 49.90; H, 4.85; N, 18.38. Found (%): C, 49.92; H, 4.86; N, 18.42. IR (ν, cm^–1^): 3415m, 3136m, 2964m, 1598s, 1548s, 1521s, 1473s, 1394s, 1352s, 1274s, 1126s, 993m, 898m, 790m, 723s, 673m, 640m, 584w, 447w.

#### 2.3.2 Synthesis of [Zn(μ-dmg)(μ-obtx)]_*n*_ (**2**)

A synthetic procedure similar to that for **1** was used except that Co(NO_3_)_2_·6H_2_O was replaced by Zn(NO_3_)_2_·6H_2_O (60 mg, 0.2 mmol). Colorless block crystals of **2** in approximately 53% (based on Zn) were washed with water and methanol and then dried in air. Anal. Calcd for C_19_H_22_N_6_O_4_Zn (463.79 g mol^–1^) (%): C, 49.20; H, 4.78; N, 18.12. Found (%): C, 49.12; H, 4.80; N, 18.15. IR (ν, cm^–1^): 3414s, 3130w, 2966m, 1606s, 1573s, 1523m, 1471w, 1388m, 1350m, 1276m, 1174w, 1128m, 995m, 900w, 792w, 723m, 673s, 640m.

#### 2.3.3 Synthesis of [Cd(μ-dmg)(μ-obtx)]_*n*_ (**3**)

A synthetic procedure similar to that for **1** was used except that Co(NO_3_)_2_·6H_2_O was replaced by Cd(OAc)_2_·2H_2_O (53 mg, 0.2 mmol). Colorless crystals of **3** in about 44% yield (based on Cd) were washed with water and methanol and then dried in air. Anal. Calcd for C_19_H_22_N_6_O_4_Cd (510.82 g mol^–1^) (%): C, 46.67; H, 4.34; N, 16.45. Found (%): C, 46.70; H, 4.36; N, 16.47. IR (ν, cm^–1^): 3415s, 3415s, 3107m, 2964w, 1539s, 1406m, 1272m, 1130s, 1008m, 987m, 898m, 721m, 673m, 644m, 607w, 580w.

#### 2.3.4 Synthesis of [Co_2_(μ-dmg)_2_(μ-pbtx)_2_]_*n*_ (**4**)

A synthetic procedure similar to that for **1** was used except that Co(NO_3_)_2_·6H_2_O was replaced by CoCl_2_·6H_2_O (48 mg, 0.2 mmol) and obtx was replaced by the pbtx ligand. Pink crystals of **4** in approximately 46% (based on Co) were washed with water and methanol and then dried in air. Anal. Calcd for C_38_H_44_N_12_O_8_Co_2_ (914.71 g mol^–1^) (%): C, 49.90; H, 4.85; N, 18.38. Found (%): C, 49.93; H, 4.88; N, 18.40. IR (ν, cm^–1^): 3448w, 3114w, 2964w, 1550s, 1475w, 1411m, 1276w, 1128m, 995w, 898w, 846w, 732m, 675m, 640w, 611w.

#### 2.3.5 Synthesis of [Cd(μ-dmg)(μ-pbtx)(H_2_O)]_*n*_ (**5**)

A synthetic procedure similar to that for **1** was used except that Co(NO_3_)_2_·6H_2_O was replaced by Cd(SO_4_)·2/3H_2_O (51 mg, 0.2 mmol) and obtx was replaced by the pbtx ligand. Colorless crystals of **5** in approximately 46% yield (based on Cd) were washed with water and methanol and then dried in air. Anal. Calcd for C_19_H_24_N_6_O_5_Cd (528.84 g mol^–1^) (%): C, 48.71; H, 4.87; N, 10.82. Found (%): C, 48.59; H, 4.92; N, 10.48. IR (ν, cm^–1^): 3448br, 1637w, 1554m, 1409w, 1278w, 1124m, 1012w, 983w, 731w, 673m, 640w, 509w.

### 2.4 Preparation of Thin Films, Including CPs

Polymeric materials play a pivotal role as matrix components in the development of optical sensor designs. In the fabrication of sensing slides, EC was selected as the supporting material due to its exceptional solvent vapor diffusibility. The EC matrix is characterized by its lack of intrinsic color/luminescence and optical transparency within the spectral range relevant to the measurements. Consequently, CPs are incorporated into the EC matrix, facilitating the unhindered diffusion of gas molecules that may react at the surface of the CP molecule. In this study, the EC-based cocktail compositions were prepared by mixing 75 mg polymer, 25 mg DOP, and 0.1 mg compounds of **1**–**5** in 2.5 mL THF. All materials were mixed in a magnetic stirrer to provide homogeneity. The optical-based measurement capabilities of gas sensors are highly dependent on the type of solid matrix and used additives. For this reason, in this study, we selected EC as a polymeric support material, which shows optical transparency, high sensitivity to NH_3_ vapor, gas permeability, and stability.

The composition of the CP-based composites is shown in Table 2. Then, the cocktails were spread onto a 125 mm polyester support (Mylar TM type) or on polycarbonate substrates by a knife coating technique. The thicknesses of the films were measured by a Tencor Alpha-Step 500 Profilometer and were found to be 5.36 ± 0.11 μm (*n* = 8). Each sensing film was cut to 1.2 cm width and fixed in the cell, and the spectra were recorded.

**Table 2:** Compositions of CP-Based Composites

sample name	compounds **1–5** (0.1 mg)	matrix (EC) (mg)	plasticizer (DOP) (mg)	solvent (THF) (mL)
1	(**1**)	75	25	2.5
2	(**2**)	75	25	2.5
3	(**3**)	75	25	2.5
4	(**4**)	75	25	2.5
5	(**5**)	75	25	2.5

## 3 Results and Discussion

### 3.1 IR Characterization of Compounds **1–5**

In this study, H_2_dmg acts as a bridging ligand in compounds **1–5**. Additionally, the contribution of the compounds obtained to their own size, pore, and topology was investigated by considering the diversity of groups that bind two triazole rings together in the molecules preferred as secondary ligands and metal salts. Five CPs containing semiflexible ligands (pbtx and obtx) and H_2_dmg aliphatic acid were systematically synthesized in distilled water with a hydrothermal method. The characteristic asymmetric and symmetric stretching vibrations corresponding to carboxylic acid groups of H_2_dmg are observed as 1694 and 1414 cm^–1^, respectively (Figure S3),^36,37^ and disappeared after compound formation.^38,39^ In all of the compounds, these stretching frequencies are observed in the region 1525–1637 cm^–1^ for υs_asym_(CO) and 1391–1476 cm^–1^ for υs_sym_(CO). In the FTIR spectra of compounds (Figures S4–S8) are observed aliphatic υ(C–H) stretching vibration ranging between 2898 and 2967 cm^–1^ and aromatic υ(C–H) stretching vibration ranging between 3130 and 3106 cm^–1^. The broad bands exhibited in the range of 3398 cm^–1^ are attributed to υ(O–H) stretching frequencies of the aqua ligands in crystal **5**.^40−42^

### 3.2 Crystal Structures of Compounds **1–5**

#### 3.2.1 [Co(μ-dmg)(μ-obtx)]_*n*_ (**1**) and [Zn(μ-dmg)(μ-obtx)]_*n*_ (**2**)

**Figure 1:**
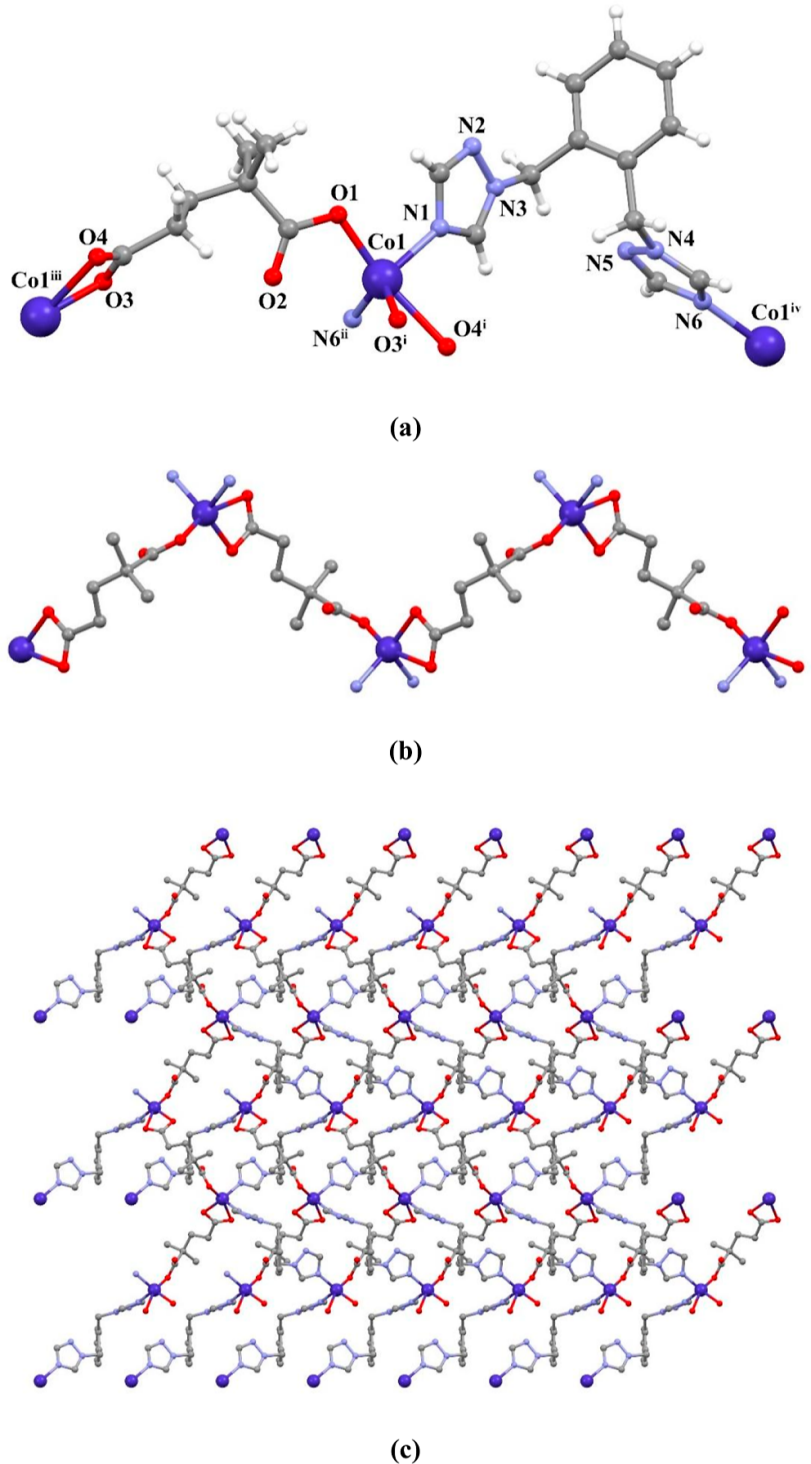
(a) Molecular structure of compound **1**, (b) zigzag 1D structure, and (c) 2D layer structure in the ab plane.

The crystal structures of compounds **1** and **2** are isomorphous. Hence, they are evaluated together. They crystallize in the monoclinic space group *P*2_1_/*n*, and the asymmetric unit contains one M(II) ion (M = Co(II) or Zn(II)), one dmg ligand and one obtx ligand (Figures 1a and S9a). The M(II) ion is five coordinated with a distorted trigonal bipyramidal geometry (/_5_ = 0.65 for **1** and /_5_ = 0.55 for **2**)^43^ by two N atoms (N1 and N6^ii^) and three O atoms (O1, O3^i^, O4^i^) from two different L ligands ((i) −*x* + 1/2, *y* + 1/2, −*z* + 3/2; (ii) −*x* + 5/2, *y* – 1/2, −*z* + 3/2 for **1** and (i) −*x* + 3/2, *y* + 1/2, −*z* + 1/2; (ii) −*x* – 1/2, *y* + 1/2, −*z* + 1/2 for **2**). The M–O/N bond lengths vary from 1.981(3) to 2.371(4) Å. These bond distances compare well to the literature values.^44,45^ The dmg ligands were coordinated to the M(II) ion from carboxylate oxygens, and the obtx ligand was coordinated from nitrogen atoms in the triazole ring as a bridging ligand. One of the carboxylate groups of the anionic dmg ligand coordinated to two different M(II) ions as a monodentate and the other as a bidentate (μ-κO:κO,O), and a zigzag-shaped 1D structure was formed (Figures 1b and S9b). The distance between the M(II) centers was measured as 8.773 Å for **1** and 8.824 Å for **2**. By connecting these 1D chains with obtx ligands, a two-dimensional (2D) layer structure was formed (Figures 1c and S9c). The obtx linkers display angular exobidentate bridging coordination modes with the intertriazole dihedral angles of 84.07° for **1** and 86.30° for **2**. The distances between the M(II) ions bridged by the obtx linker are 11.023 Å for **1** and 11.002 Å for **2**. The 3D supramolecular structure of the compound is formed by the interactions of π···π, C–H···π, and C–H···O.

#### 3.2.2 [Cd(μ-dmg)(μ-obtx)]_*n*_ (**3**)

**Figure 2:**
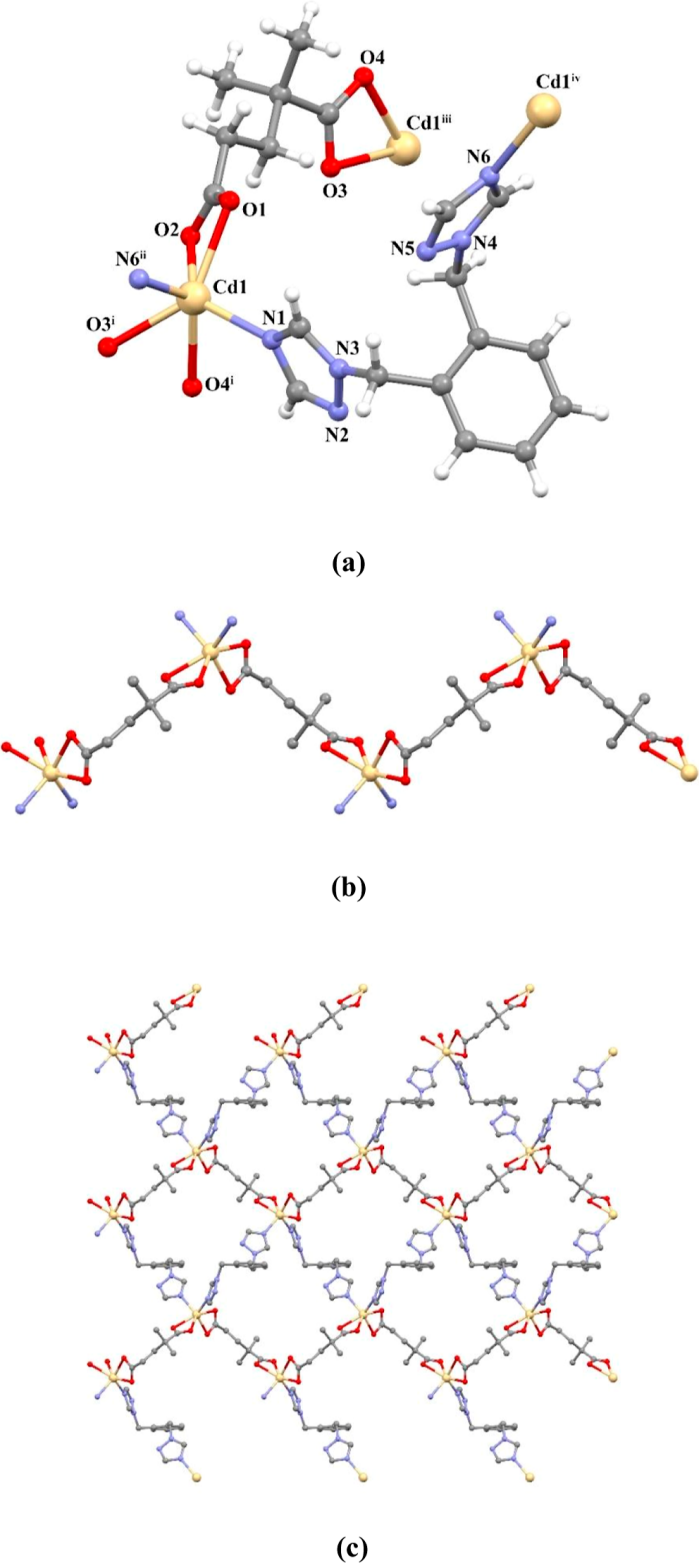
(a) Molecular structure of compound **3**, (b) zigzag 1D structure, and (c) 2D layer structure in the ab plane.

An X-ray structural analysis demonstrates that **3** crystallizes in the monoclinic system with a *P*2_1_/*n* space group. The asymmetric unit contains one cadmium(II) center, one dmg ligand, and one obtx ligand. Each Cd(II) ion in **3** exhibits a distorted octahedral coordination environment, composed of chelated four carboxylic O atoms from two dmg ligands and coordinated two N atoms from two obtx ligands, as shown in Figure 2a. The carboxylate groups of the anionic dmg ligand coordinated to two different Cd(II) ions as bis(bidentate) (μ-κO,O:κO,O), and a zigzag-shaped 1D polymeric structure was formed (Figure 2b). The distance between the Cd(II) centers was measured as 9.054 Å. By connecting these 1D chains with obtx ligands, a 2D layer structure was formed (Figure 2c). The dihedral angle between the triazole rings in the neutral obtx ligand is 87.95°. Additionally, the distance between the Cd(II) ions bridged with the obtx ligand was measured as 11.167 Å. Cd–N and Cd–O bond lengths range from 2289(2)–2360(2) Å. The 3D supramolecular structure of the compound was formed by the interactions C–H···π and C–H···O.

#### 3.2.3 [Co_2_(μ-dmg)_2_(μ-pbtx)_2_]_*n*_ (**4**)

**Figure 3:**
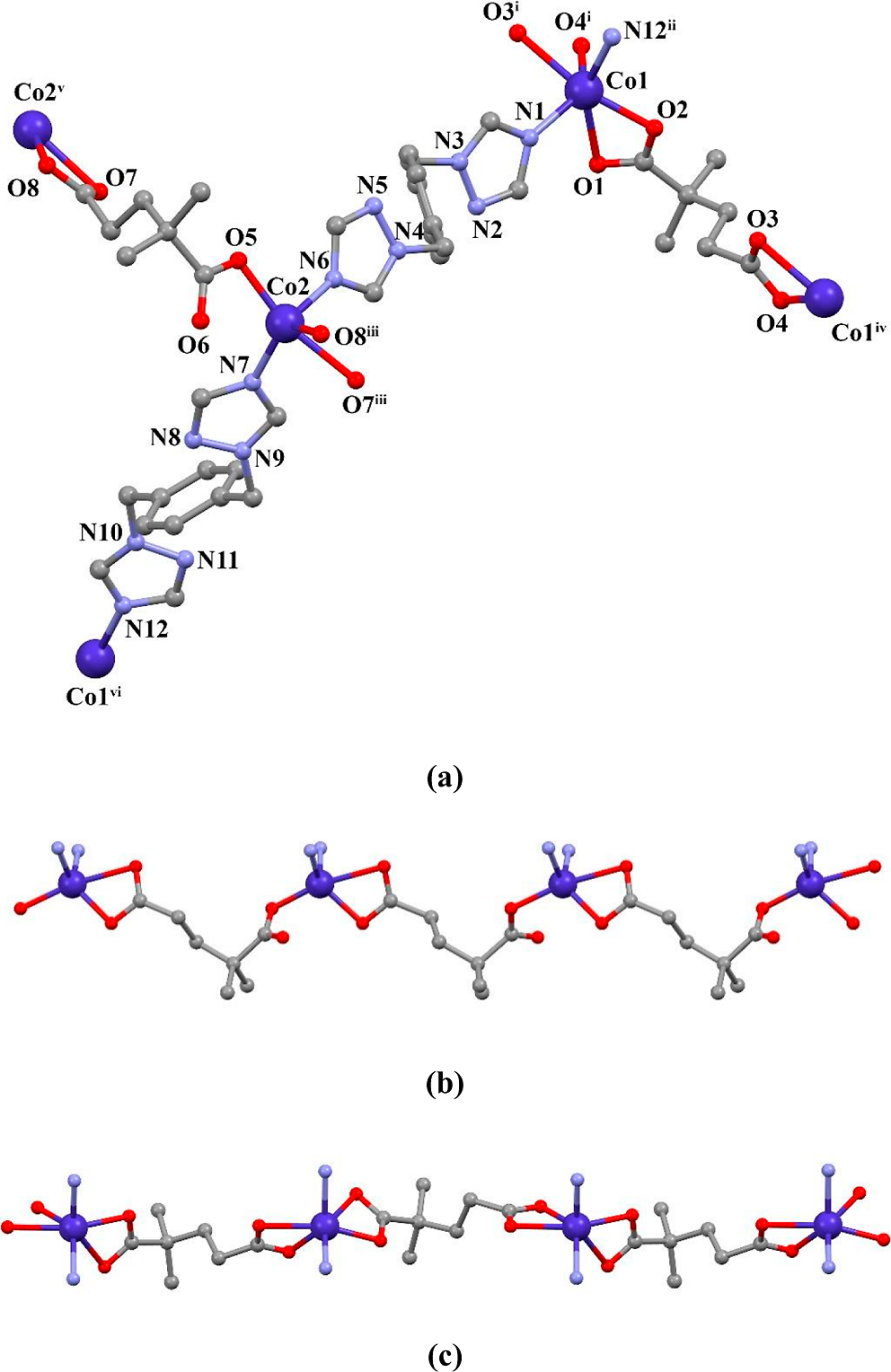
(a) Molecular structure of compound **4**, (b) 1D structure, and (c) other 1D structures.

**Figure 4:**
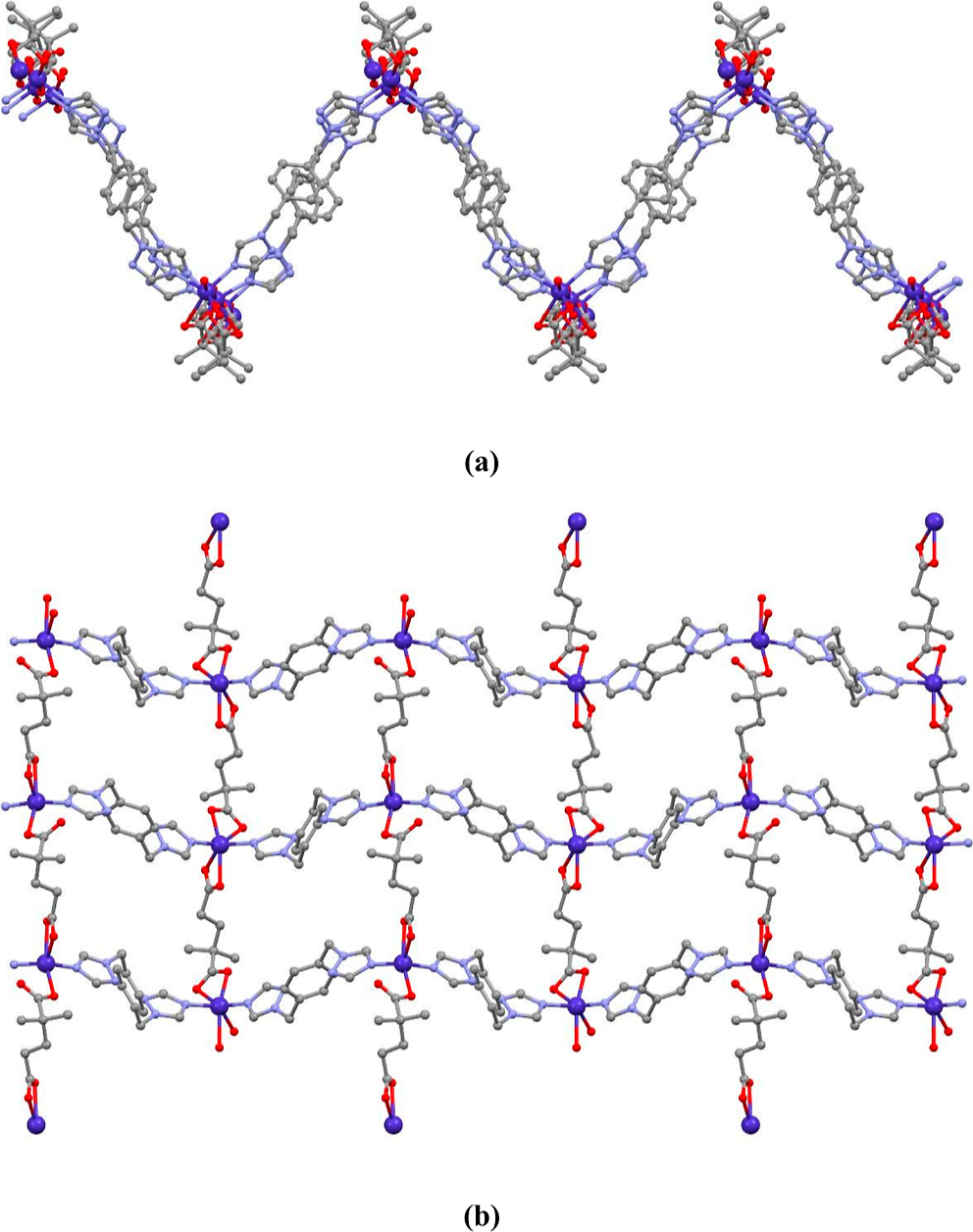
(a) 2D wavy structure of compound **4** in the bc plane and (b) 2D wavy structure in the ab plane.

Compound **4** was synthesized by the reaction of cobalt(II) chloride with H_2_dmg and pbtx in H_2_O at 100 °C. SCXRD structural analysis reveals that **4** crystallizes in the orthorhombic space group *Pca*2_1_. As shown in Figure 3a, there are two Co(II) centers, two dmg ligands, and two pbtx ligands in the asymmetric unit of the compound. The Co1 ion in the compound is six-coordinated and has a distorted octahedral geometry. The carboxylate oxygens of two different dmg ligands and the nitrogen atoms of two different pbtx ligands were coordinated to the Co1 ion. The Co2 ion is five-coordinated with a distorted trigonal bipyramidal geometry (/_5_ = 0.61). Two different dmg ligands with three carboxylate oxygen atoms and pbtx ligands with two nitrogen atoms were coordinated to the Co2 ion. The anionic dmg ligands coordinate to the metal center through the monodentate and bidentate (μ-κO:κO,O) and bis(bidentate) (μ-κO,O:κO,O) coordination modes. Two cobalt(II) centers are connected by dmg ligands to form two different 1D flat polymeric chains (Figure 3b,c). A 2D wavy structure was formed by connecting these 1D chains with pbtx ligands (Figure 4a,b). The dihedral angles between the triazole rings located on the neutral pbtx ligands are 12.05° (with N1–N3–N4–N6) and 12.38° (with N7–N9–N10–N12). Additionally, the distances between the pbtx ligands and the bridged Co(II) ions were measured as 14.996 and 15.337 Å. The 3D supramolecular structure of the compound was formed by the interactions of C–H···π and C–H···O.

#### 3.2.4 [Cd(μ-dmg)(H_2_O)(μ-pbtx)]_*n*_ (**5**)

**Figure 5:**
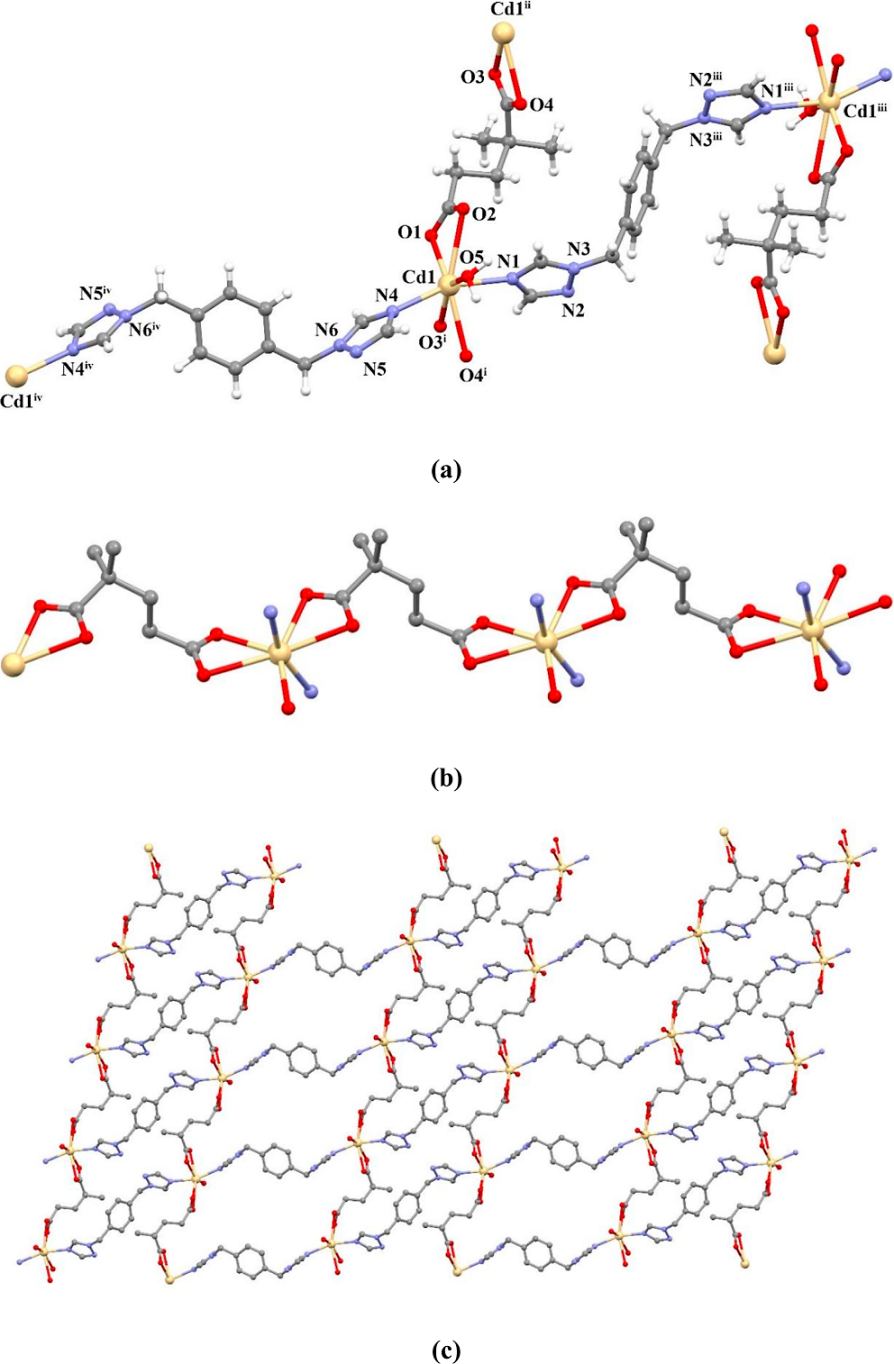
(a) Molecular structure of compound **5**, (b) 1D structure, and (c) 2D layer structure in the ac layer.

**Figure 6:**
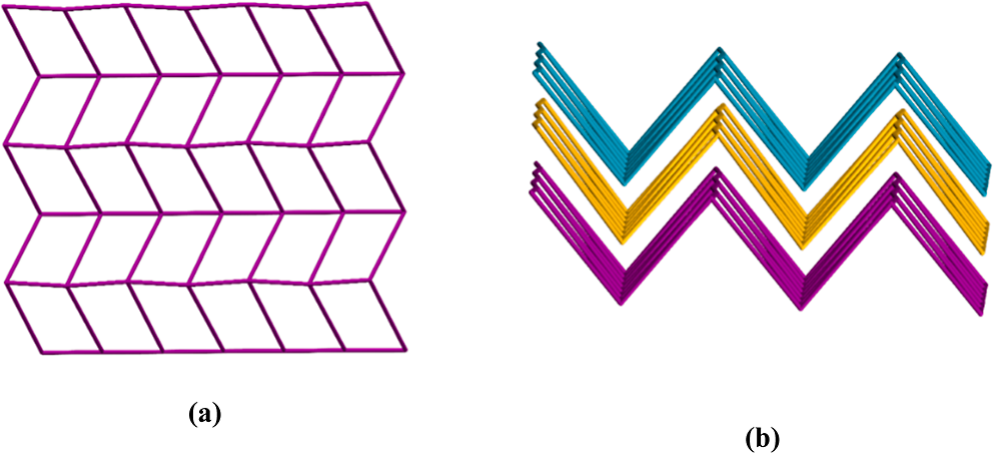
(a) Schematic view of the 4-connected 2D network of all compounds along the ab plane and (b) 3D supramolecular network of all compounds along the bc plane.

**Figure 7:**
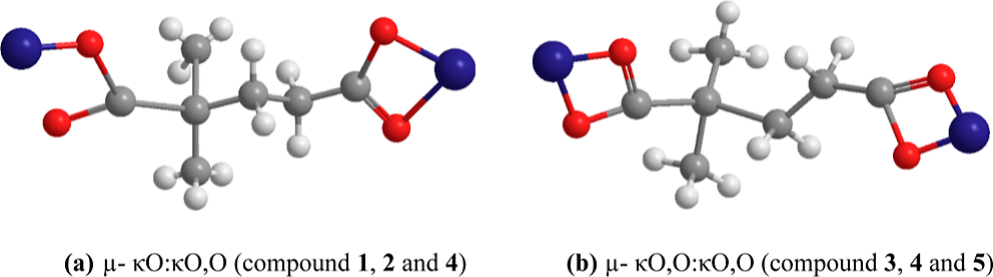
Diverse coordination modes of dmg^2–^ in compounds **1–5**.

Compound **5** was formed following the reaction of cadmium(II) sulfate with H_2_dmg and pbtx in H_2_O at 100 °C. It crystallizes in the triclinic space group *P*1̅, and the asymmetric unit contains one crystallographically independent Cd(II) center, one dmg, two different half pbtx ligands, and one aqua ligand. Each Cd(II) ion in **5** exhibits a distorted pentagonal bipyramidal coordination environment, composed of chelated four carboxylic O atoms from two L ligands, coordinated one O atom from the aqua ligand in the equatorial direction, and coordinated two N atoms from two pbtx ligands in the apical direction, as shown in Figure 5a. The dmg ligand behaved as a bis(bidentate) (μ-κO,O:κO,O) ligand by bridging two Cd(II) ions. A 1D polymeric chain structure was formed by the dmg ligand, forming a bridge between two Cd(II) ions (Figure 5b). By connecting these 1D chains with pbtx ligands, a 2D layer structure was formed (Figure 5c). The dihedral angles between the triazole rings located on the pbtx ligands are 0°. The distance between the Cd(II) ions bridged by the pbtx ligand was measured as 15.185 Å. The Cd–N and Cd–O bond lengths change in the range of 2.2670(18)–2.5473(17) Å. These values are also compatible with similar structures found in the literature.^44^ The 3D supramolecular structure of the compound was formed by the interactions of C–H···π and C–H···O. According to the topological analysis of all compounds, 2D structures consisting of 4-connected metal centers have sql topologies with the point symbol {4^4^.6^2^} (Figure 6). In addition, the binding modes of the dmg^2–^ ligand found in the structure of all compounds are given in Figure 7.

### 3.3 PXRD and Thermogravimetric Analysis of Compounds **1–5**

To check the phase purity of compounds **1**–**5**, PXRD analyses were carried out at room temperature. As shown in Figures S10–S14, the peak positions of the experimental and simulated PXRD patterns of the compounds were in agreement with each other, demonstrating the good phase purity of the compounds. TGA curves for **1–5** were recorded to investigate the thermal stabilities of the compounds in a static air atmosphere in the temperature range of 30–700 °C as shown in Figures S15–S19. All of the compounds showed stability up to 300 °C apart from compound **5**. The anhydrous compounds generally were decomposed in two steps.

For compound **1**, the first stage began to decompose at 239 °C. The weight loss of 29.2% was attributed to the removal of the dmg ligand (calcd. 26.3%). The weight loss of 34.7% from 353 to 410 °C can be ascribed to the release of obtx (calcd. 34.5%). For compound **2**, the first weight loss of 31.3% in the region of 285–317 °C (calcd. 34.5%) corresponds to the loss of the dmg ligand. It began to decompose beyond 482 °C with the second weight loss of 18.4% in the region of 482–506 °C (calcd. 26.2%) corresponding to the loss of the obtx ligand. For compound **3**, the weight loss of 32.7% in the temperature range of 291–331 °C was consistent with the removal of dmg (calcd. 37.1%). The weight loss corresponding to the release of the obtx ligand was observed from 512 to 562 °C. For compound **4**, the first step of weight loss occurred between 310 and 331 °C, which attributed to the loss of dmg, with a weight loss of 22.6% in agreement with the calculated 34.5% and the collapse of the framework. Above 280 °C, a successive weight loss of 31.2% was observed above 360 °C, which corresponds to the liberation of the pbtx ligand (calcd. 26.5%). For compound **5**, the first weight loss corresponds to the release of the aqua ligand at around 150 °C (obsd. 1.98%, calcd. 1.74). The second weight loss of 5.96% between 268 and 316 °C corresponds to half of the pbtx ligand (calcd. 5.60%). In the third step, a loss of 6.89% was observed in the temperature range of 491–627 °C, which was attributed to the release of another half of the pbtx ligand (calcd. 6.86%). The residue corresponds to the formation of metal oxides CoO (obsd. 18.27%, calcd. 16.4%) for **1**, ZnO (obsd. 20.34%, calcd. 17.5%) for **2**, CdO (obsd. 24.5%, calcd. 25.0%) for **3**, CoO (obsd. 29.2%, calcd. 16.4%) for **4**, and CdO (obsd. 27.55%, calcd. 24.78%) for **5**.

### 3.4 Performance Tests of Compounds **1–5** against NH_3_ Vapor

**Figure 8:**
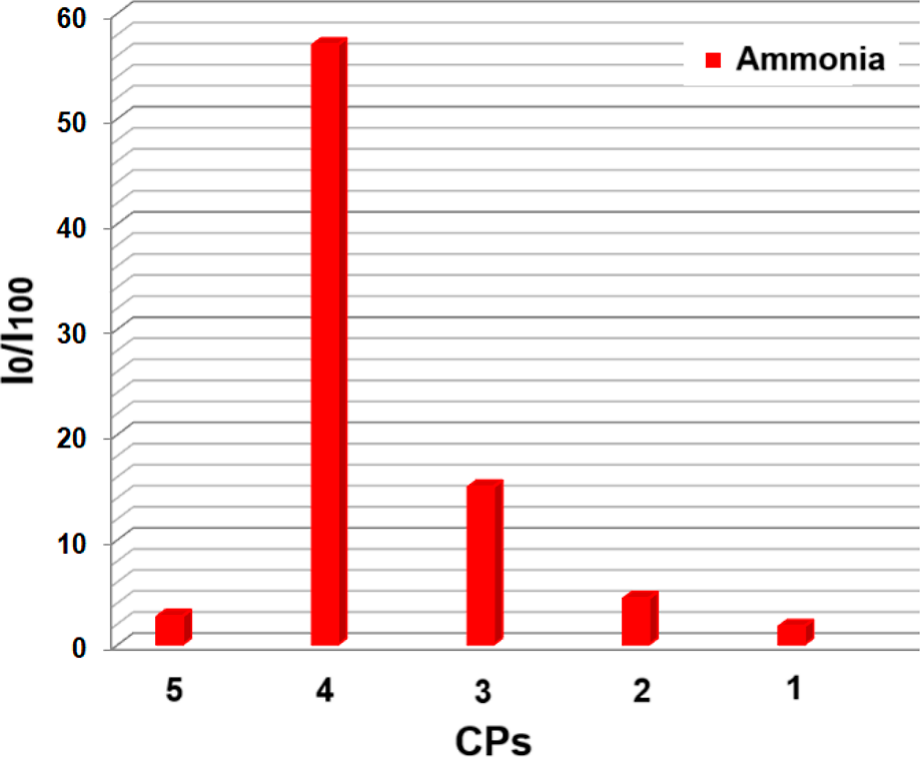
Detection sensitivity was measured after exposure to NH_3_ vapor.

In this study, the performance tests of the synthesized compounds against NH_3_ vapor were carried out by exposing thin films in which the **1–5** compounds were embedded in the EC polymeric matrix, respectively, to different concentrations of NH_3_ vapor in the desiccator. The prepared CP-based polymeric sensor agents were placed in a 100 mL beaker filled with 50 mL of NH_3_ solvent and incubated in the dark for 60 min in a vacuum desiccator. Vapor was obtained by the evaporation of NH_3_ solvent under laboratory conditions. The average laboratory temperature was measured as 20 °C (±0.4 °C) throughout the experiments. The concentrations of NH_3_ vapor were determined based on its respective partial vapor pressures at 20 °C and ammonia (NH_3_, 25% v/v solution) at 40.32 kPa. The partial pressures of solvent vapor were further expressed in parts per million (ppm) for a comprehensive analysis. The NH_3_ sensitivity (*I*_0_/*I*_100_) of CP-based sensing agents were plotted in Figure 8 as relative signal changes, where *I*_100_ is the fluorescence intensity of the sensor membrane after exposure to NH_3_ vapor exposed for 500 ppm and *I*_0_ is the fluorescence intensity of the sensor slide in 0 ppm of NH_3_ vapor.

**Figure 9:**
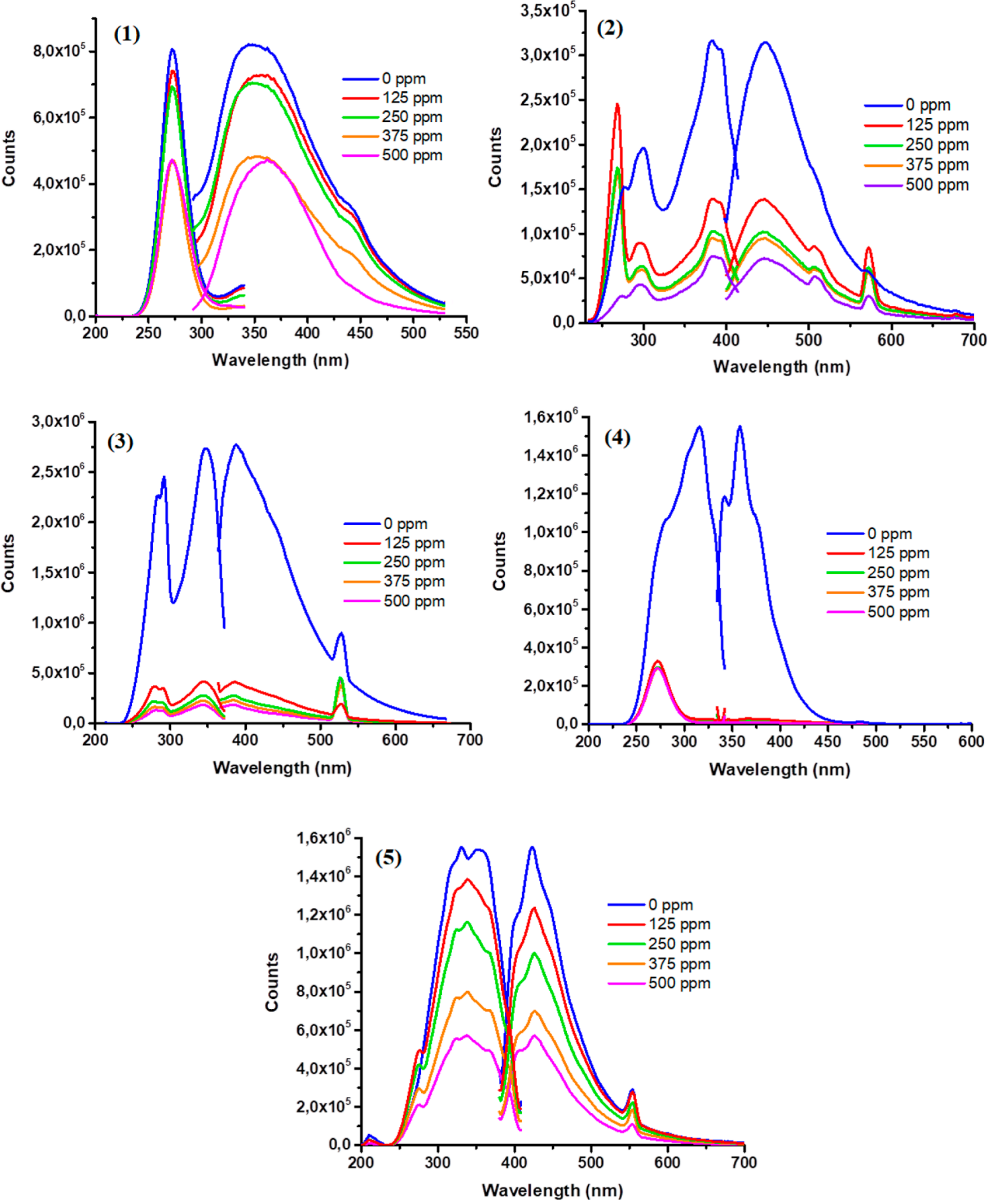
Excitation and emission spectra of CP-based thin films exposed to NH_3_ vapor for 0, 15, 30, 45, and 60 min, respectively: (1) **1**, (2) **2**, (3) **3**, (4) **4**, and (5) **5**.

However, it was determined that the most sensing slides against NH_3_ vapor among CP-based composites were the **4**-based thin film (Figure 9). However, the limit of detection (LOD-3*s*/*k*) was calculated with *s* being the standard deviation of the blank and *k* denoting the slope between intensity and CP concentration, and the LOD of **4** was determined as 8.9 μM.

The CP-based sensing composites used are sensitive to quenching by NH_3_ molecules. Quantitative measurement of fluorescence quenching in a homogeneous medium can be performed by calculating the Stern–Volmer constant (*K*_SV_) (see eq 1)
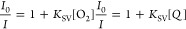
1where *I*_0_ and *I* are the emission intensities in the absence and presence of a quencher, respectively. *K*_SV_ is the Stern–Volmer constant. [*Q*] is the concentration of the extinguisher in the environment. According to the equation, the *I*_0_/*I* value increases in direct proportion to the concentration of the quencher. When all other variables are constant, the higher the *K*_SV_ is, the lower the quencher concentration required to quench the fluorescence. The emission and excitation measurements of CP-based compounds embedded in thin films exposed to different concentrations of NH_3_ vapors were measured using a photoluminescence (PL) spectrophotometer and were shown in Figure 9. In the PL measurements taken against the NH_3_ concentration, a decrease in the intensity values of all compound-containing detection agents was observed depending on the increase in the amount of NH_3_ vapor. The concentrations of NH_3_ vapor to which the CP-based composites are exposed were calculated using the solvent partial vapor pressure (NH_3_ (25% v/v) 40.32 kPa) and the solvent evaporation rate at 20 °C. Calculated ppm concentrations of NH_3_ vapor exposed against the time of sensor agents embedded in the EC thin film phase are given in Table 3.

**Table 3:** Calculated ppm Concentrations of NH_3_ Vapor Exposed against the Time of Sensor Agents Embedded in the EC Thin Film

compound name	λ_ex1_	λ_ex2_	λ_ex3_	λ_em1_	λ_em2_	λ_em3_	*I*_0_/*I*_100_	NH_3_ (ppm)				
								0 min	15 min	30 min	45 min	60 min
**1**	275			350			1.75	0	125	250	375	500
**2**	268	300	385	445	505	570	4.40	0	125	250	375	500
**3**	290	350		390	530		15.00	0	125	250	375	500
**4**	270	320		360			57.00	0	125	250	375	500
**5**	350			425	555		2.70	0	125	250	375	500

Upon excitation at 275, 268, 290, 270, and 350 nm of compounds **1**–**5**, the thin films exhibited several emission peaks between 300 and 600 nm (see Table 3). The CPs containing EC-based thin films were exposed to NH_3_ vapor for a duration of 60 min under laboratory conditions. Upon exposure, both excitation and emission spectra of the prepared thin films showed significant signal reduction, as illustrated in Figure 9. The results were quantified as relative fluorescence changes using the algorithm (*I*_0_/*I*_100_) for the *y*-axis, where *I*_0_ and *I*_100_ represent the fluorescence intensities in the absence and presence of the quencher, respectively. The gas sensitivities (*I*_0_/*I*_100_) of CP-based sensing materials were determined as 1.75, 4.40, 15.00, 57.00, and 2.70 when exposed to NH_3_ for compounds **1–5**, respectively. The incorporation of CP additive into the EC as thin films yielded numerous benefits, including heightened sensitivity and advancements in the overall dynamics of optical gas sensing.

### 3.5 Optical Gas Sensing Mechanism

In this study, we explored the gas-sensing capabilities of CPs when they are exposed to NH_3_ vapor. CPs are materials with repeating coordination bonds between metal ions or clusters and organic ligands. These materials often exhibit interesting optical properties that can be exploited for gas-sensing applications. CP-based composites offer advantages such as tunable properties and high surface area, making them promising candidates for gas-sensing applications, including the detection of ammonia. As can be seen, the emission intensities of the materials under study were quenched with increasing exposure quantity to solvent vapors. The quenching obtained in PL-based measurements has been the most widely used form of signal transduction when MOFs are exposed to VOCs. The nature of host–guest interactions affects the strength of PL quenching. Usually, the related interactions occur on the basis of the electron donor/electron acceptor orbital overlap phenomenon. Strong electron-withdrawing functional groups such as nitroaromatic compounds and nitro groups are among the analytes that are easily detected by CPs/MOFs. Thus, the resulting quenching mechanism can be attributed to the electron transfer from electron-donating MOFs in the excited state to electron-withdrawing nitroaromatic compounds.^46^ Recently, CPs based on obtx and pbtx linkers, which have excellent electron-accepting abilities, have become very popular.^47^ The optical gas sensor mechanism for CPs responding to NH_3_ vapor involves the coordination of ammonia with metal centers and ligands, leading to changes in the electronic structure and optical properties of the CP. Since ammonia is a good electron donor and a Lewis base, the addition of pbtx linkers to the coordination materials can also impart selectivity for the visual detection of amines. The addition of pbtx linkers to coordination materials modifies the chemical composition and structure of the CP. These linkers likely have specific binding sites that can interact with ammonia. Accordingly, it was determined that the **4**-based thin films were the most sensitive among the CP-based composites used to detect ammonia. The [Co_2_(μ-dmg)_2_(μ-pbtx)_2_]_*n*_ CP, with its specific ligands (dmg and pbtx), likely has a selective affinity for ammonia molecules. The chosen ligands, especially pbtx, may facilitate a strong and selective interaction with ammonia, contributing to increased sensitivity. It has been observed that the purple color of the Co (II) ion-based **4** detection agent, which can detect ammonia with color changes with the naked eye, turns blue when exposed to ammonia vapor (Figure S20). The metal centers (Co) and ligands in the CP can participate in coordination bonding and electron transfer with ammonia. This interaction induces changes in the electronic structure of the CP, leading to detectable alterations in its optical properties. The reason for the color change mechanism caused by ammonia is thought to be due to the formation of free radicals in pbtx binders. When exposed to ammonia vapor, ammonia molecules can act as electron donors to donate electrons to the—pbtx ligand to form—pbtx free radicals involved in the **4**-sensing agent. Since pbtx ligands bind to the benzene ring from the para-position, electron delocalization is thought to affect the longer chain. Quite different results were observed for ammonia adsorption when the **4** agent was compared with **1**, which also has the Co (II) ion in the center but has a different ligand, obtx. In the presence of NH_3_ molecules acting as electron donors, electron delocalization remained in a restricted area due to bonding from the ortho-position of CP-based composites containing the obtx ligand. Therefore, CP-based composites with the obtx ligand were not sensitive to NH_3_ vapor. Two novel multichromatic CPs based on a new flexible viologen ligand exhibiting photocontrolled luminescence properties and sensitive detection for ammonia. In addition, to determine the stability of **4@NH**_**3**_, PXRD analysis was performed after **4** was exposed to ammonia for 15 and 60 min. According to the results of the analysis, it was determined that the PXRD patterns of **4** before and after ammonia exposure were compatible and **4** was stable (Figure S21).

## 4 Conclusions

The formation and characterization of five new 2D CPs with semiflexible bis(triazole) ligands and aliphatic dicarboxylic acids were carried out. Compounds **1–5** exhibited a 2D framework. Topologically, all compounds are 2-nodal 4-connected 2D networks and display sql topologies with a point symbol of {4^4^.6^2^}. This study revealed that the usage of dmg, obtx, and pbtx ligands was an effective way to construct new functional CPs. The compounds **1**, **2**, and **3** were isostructural and displayed flat 2D networks. When the obtx ligand is replaced with the pbtx ligand and in the presence of Co(II), compound **4** displays an undulated 2D network. The framework of compounds collapses around 300 °C apart from compound **5** that has an aqua ligand. The anhydrous compounds generally decompose in two steps. Compound **4** displayed sensitive and selective detection toward NH_3_ vapor through luminescence quenching in the presence of other interference agents. As a result, compound **4** could be used as a luminescent sensor for the detection of ammonia vapor.

## Supporting Information Available

Crystallographic data for the structural analysis have been deposited with the Cambridge Crystallographic Data Centre, CCDC no. 2179646 for **1**, 2179647 for **2**, 2179648 for **3**, 2179649 for **4**, and 2179650 for **5**. Copies of this information may be obtained free of charge from the Director, CCDC, 12 Union Road, Cambridge CB2 1EZ, UK (fax: +44-1223-336033; e-mail: deposit@ccdc.cam.ac.uk or www: http://www.ccdc.cam.ac.uk)
Crystallographic data for complex **1** (CIF)
Crystallographic data for complex **2** (CIF)
Crystallographic data for complex **3** (CIF)
Crystallographic data for complex **4** (CIF)
Crystallographic data for complex **5** (CIF)
^1^H NMR spectrum of pbtx and obtx ligand; IR spectrum of 2,2-dimethylglutaric acid, **1**, **2**, **3**, **4**, and **5**; molecular structure of complex **2**, zigzag 1D structure, and 2D layer; PXRD pattern of **1**, **2**, **3**, **4**, and **5**; thermal analysis curves of **1**, **2**, **3**, **4**, and **5**; PXRD pattern of **4** and **4@NH**_**3**_; selected bond distance and angle data for **1***, **2***, **3***, **4***, and **5*** (PDF)

## Supplementary Material

ao3c03212_si_001.cif

ao3c03212_si_002.cif

ao3c03212_si_003.cif

ao3c03212_si_004.cif

ao3c03212_si_005.cif
