# Supplementary material for: Sensitivity to NH3 Vapor: Synthesis and Characterization of Five New Coordination Polymers Based on 2,2-Dimethylglutaric Acid and Bis(triazole)-Derived Ligands
Source: ACS Omega. 2024 Jan 8;9(3):3193–203. doi: 10.1021/acsomega.3c03212 (PMC10809242; doi:10.1021/acsomega.3c03212)
Supplement: Supplementary file 6 — ao3c03212_si_006.pdf [file ao3c03212_si_006.pdf]

Sensitivity to NH_3 Vapor: Synthesis and Characterization of Five New Coordination Polymers based on 2,2-Dimethylglutaric Acid and Bis(triazole) Derived Ligands

Pelin Köse Yaman[a*], Özge Demir[b], Sevde Demir[b], Merve Zeyrek Ongun[c], Sibel Oğuzlar[d], and Hakan Erer[b*]

[a] Dokuz Eylül University, Faculty of Science, Department of Chemistry, İzmir, Türkiye

[b] Eskişehir Osmangazi University, Faculty of Science, Department of Chemistry, Eskişehir, Türkiye

[c] Dokuz Eylül University, Chemistry Technology Program, İzmir Vocational High School, İzmir, Türkiye

[d] Dokuz Eylül University, Center for Fabrication and Application of Electronic Materials, İzmir, Türkiye

*Corresponding authors. Fax: +90 2324534188 (P. Köse Yaman), +90 2222393578 (H. Erer)

E-mail address: pelin.kose@deu.edu.tr (P. Köse Yaman), herer@ogu.edu.tr (H. Erer)

Table of contents

Fig. S1. The ^{1}H-NMR spectrum of pbtx ligand 3
Fig. S2. The ^{1}H-NMR spectrum of obtx ligand 3
Fig. S3. IR spectrum of 2,2-dimethylglutaric acid (H_2dmg) 4
Fig. S4. IR spectrum of **1** 4
Fig.S5. IR spectrum of **2** 5
Fig. S6. IR spectrum of **3** 5
Fig. S7. IR spectrum of **4** 6
Fig. S8. IR spectrum of **5** 6
Fig. S9. **(a)** Molecular structure of complex **2**, **(b)** zigzag 1D structure, **(c)** 2D layer structure 7
Fig. S10. PXRD pattern of **1** 8
Fig. S11. PXRD pattern of **2** 8
Fig. S12. PXRD pattern of **3** 9
Fig. S13. PXRD pattern of **4** 9
Fig. S14. PXRD pattern of **5** 10
Fig. S15. Thermal analysis curves of **1** 11
Fig. S16. Thermal analysis curves of **2** 12
Fig. S17. Thermal analysis curves of **3** 13
Fig. S18. Thermal analysis curves of **4** 14
Fig. S19. Thermal analysis curves of **5** 15
Fig. S20. PXRD pattern of **4** and **4@NH_3.** 16
Table S1. Selected bond distance (Å) and angle (°) data for **1*** 17
Table S2. Selected bond distance (Å) and angle (°) data for **2*** 18
Table S3. Selected bond distance (Å) and angle (°) for **3*** 19
Table S4. Selected bond distance (Å) and angle (°) for **4*** 20
Table S5. Selected bond distance (Å) and angle (°) for **5*** 21

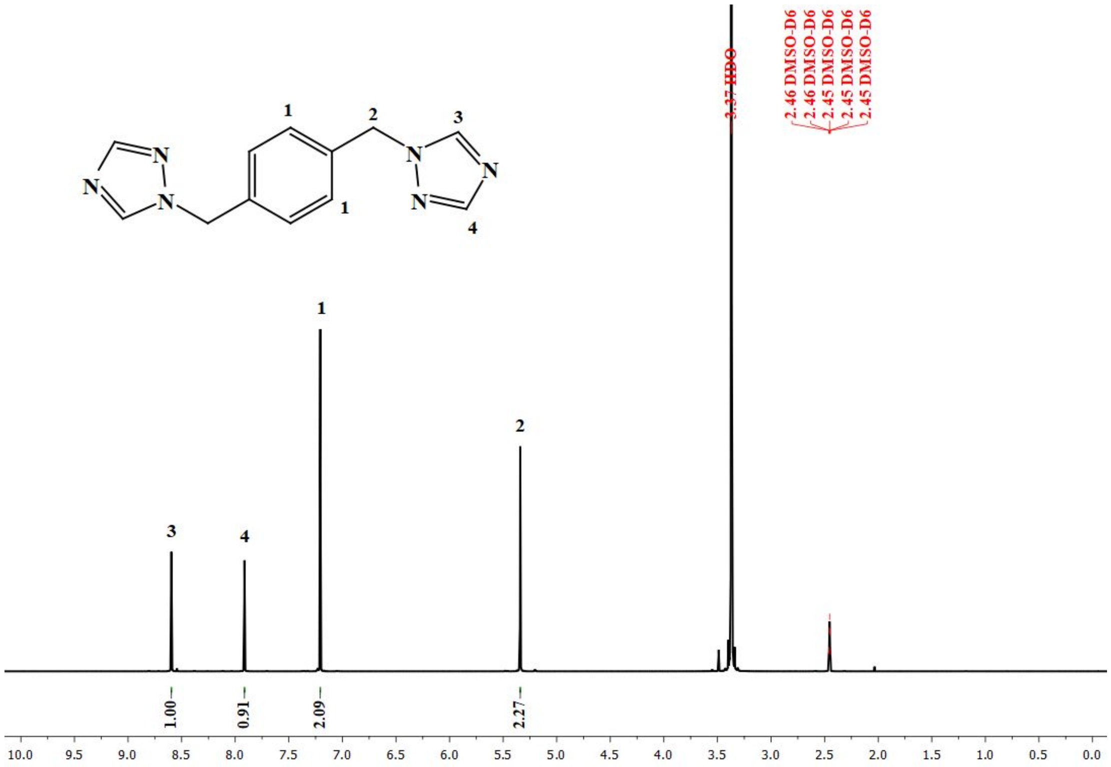

Fig. S1. The ^{1}H-NMR spectrum of pbtx ligand

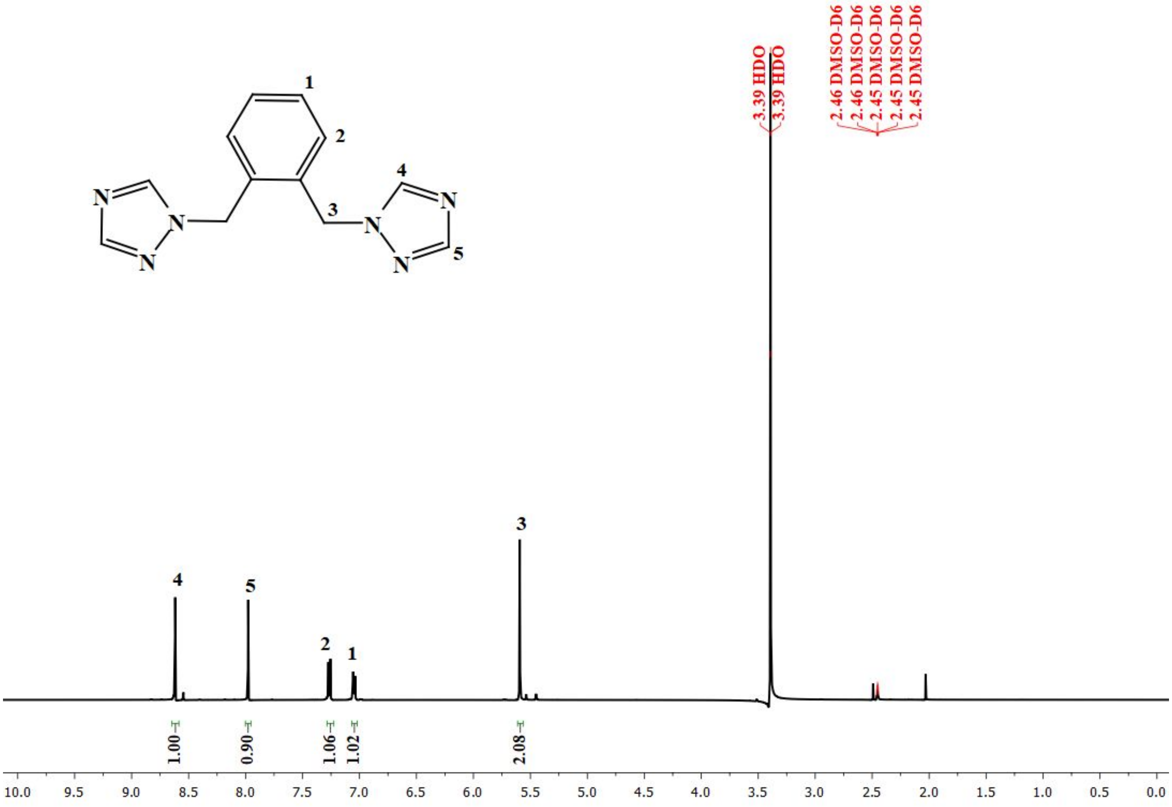

Fig. S2. The ^{1}H-NMR spectrum of obtx ligand

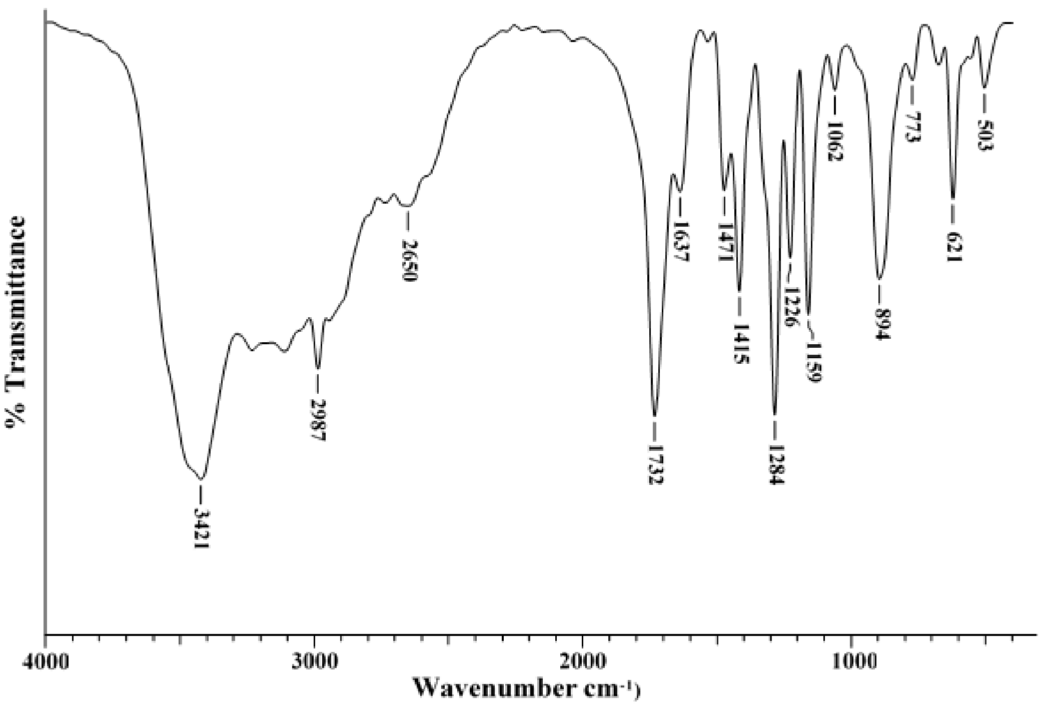

Fig. S3. IR spectrum of 2,2-dimethylglutaric acid (H_2dmg)

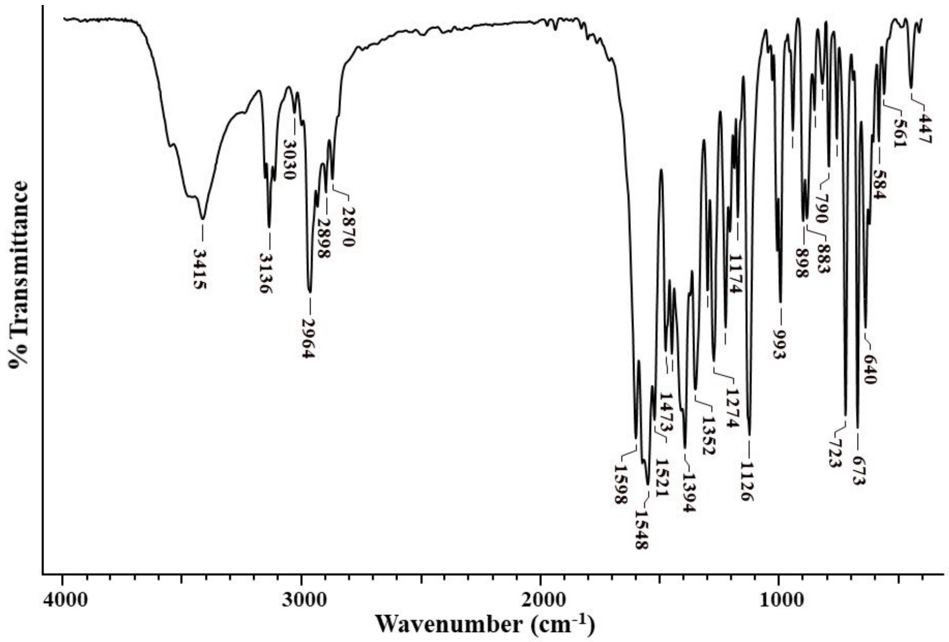

Fig. S4. IR spectrum of **1**

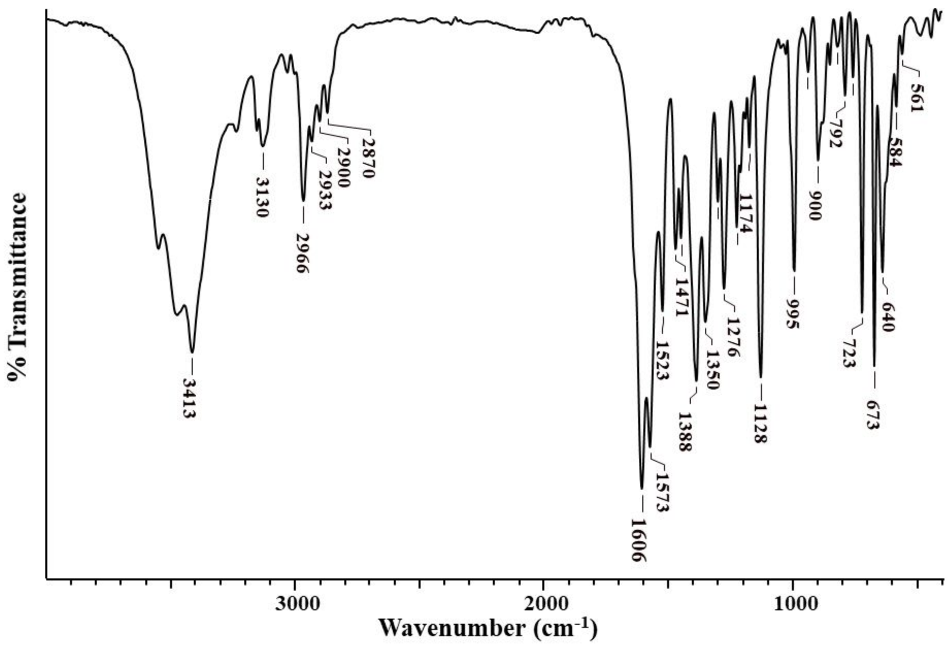

Fig.S5. IR spectrum of **2**

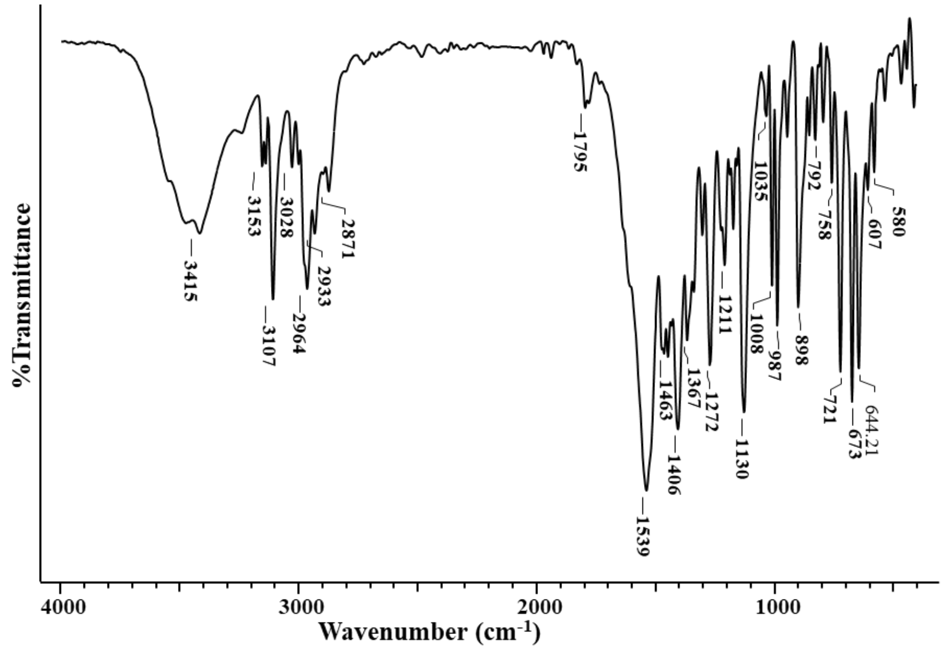

Fig. S6. IR spectrum of **3**

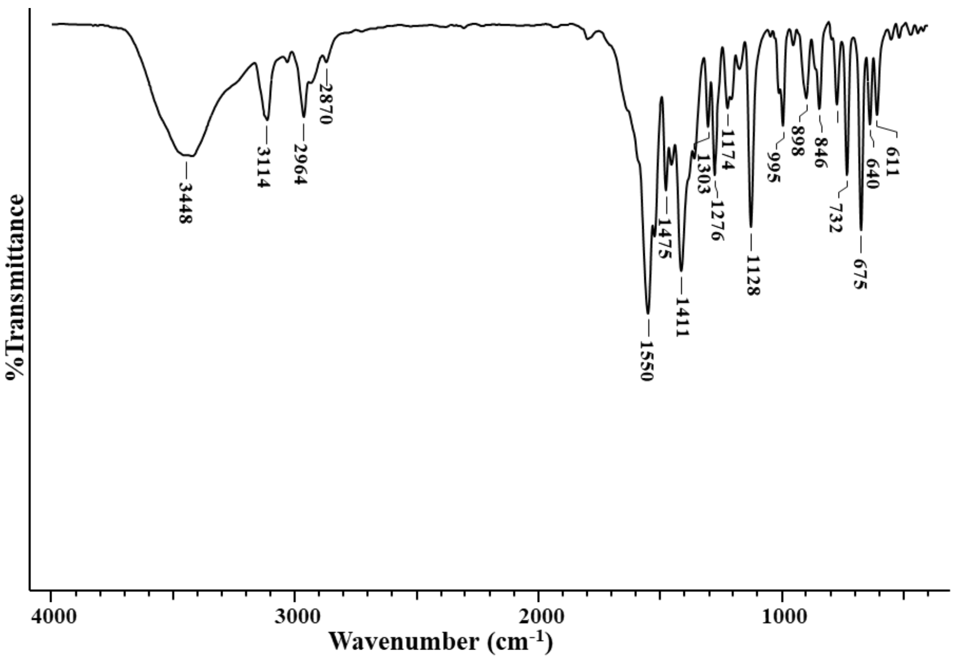

Fig. S7. IR spectrum of **4**

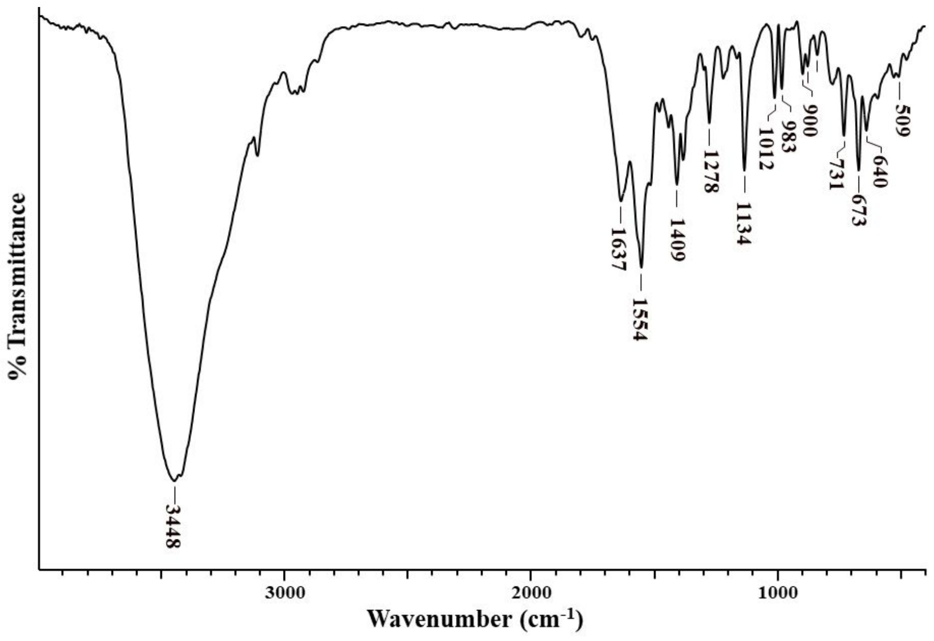

Fig. S8. IR spectrum of **5**

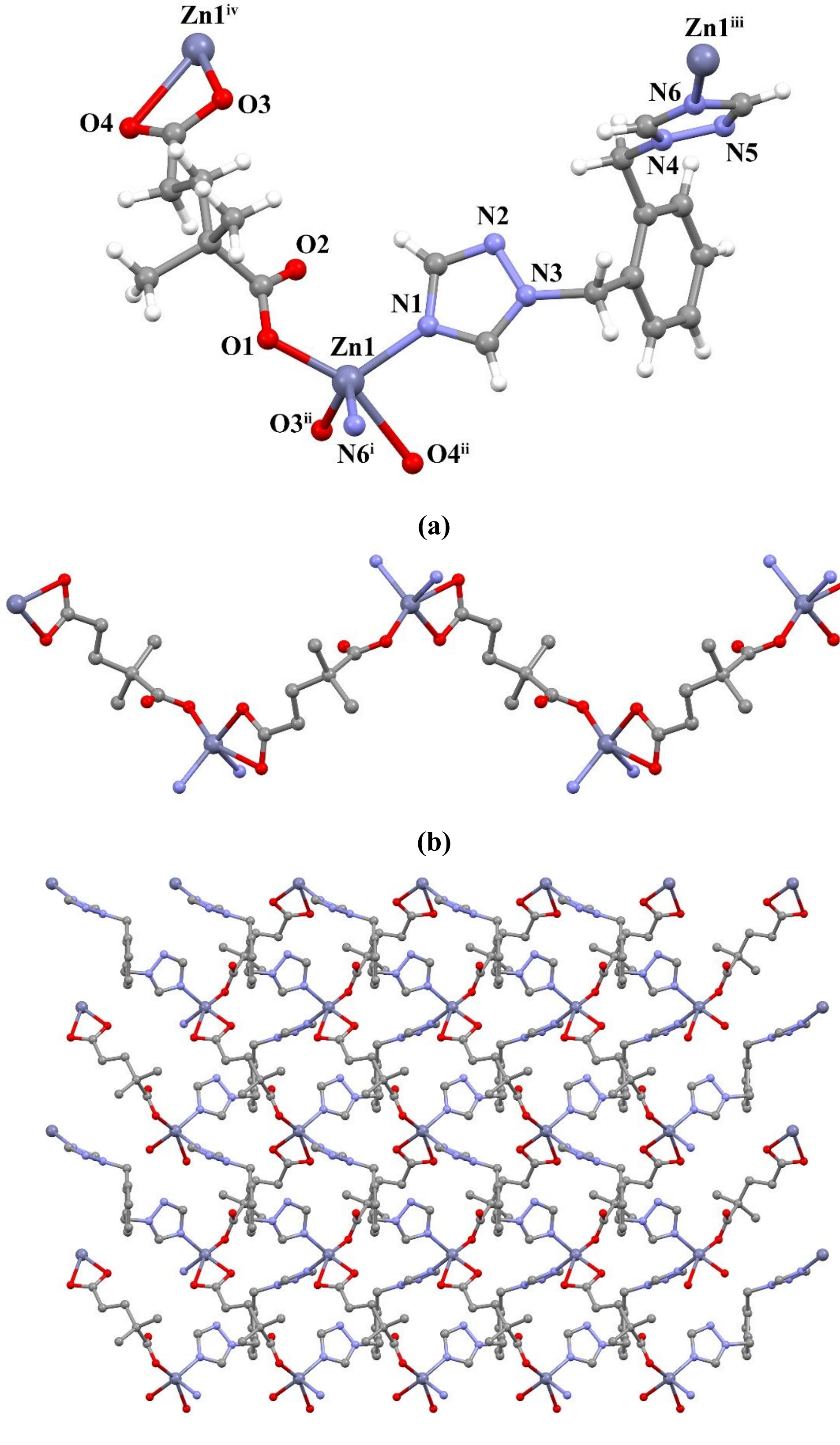

(c)

Fig. S9. **(a)** Molecular structure of complex **2**, **(b)** zigzag 1D structure, **(c)** 2D layer structure

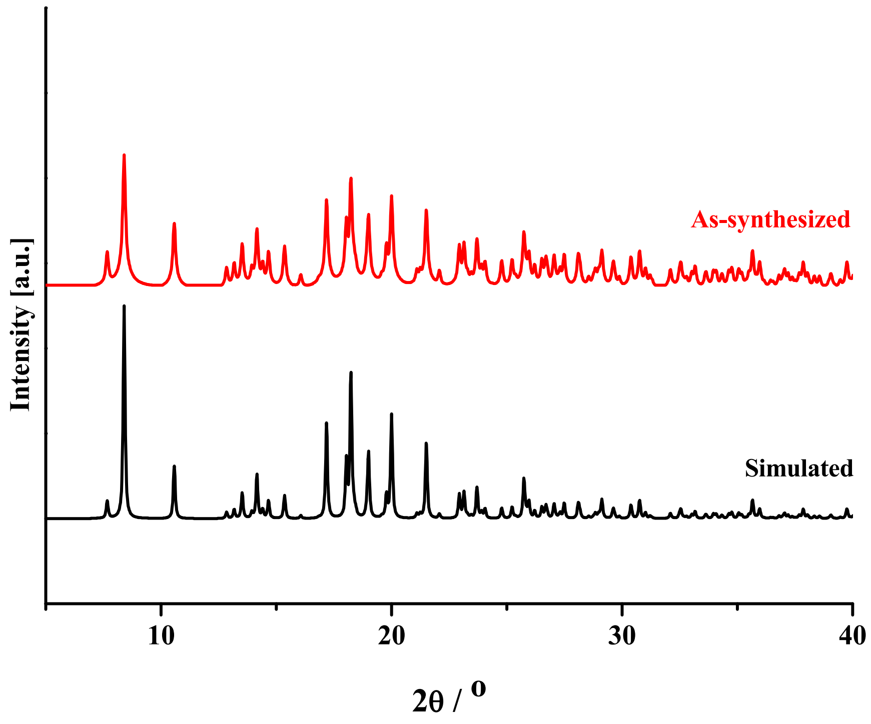

Fig. S10. PXRD pattern of **1**

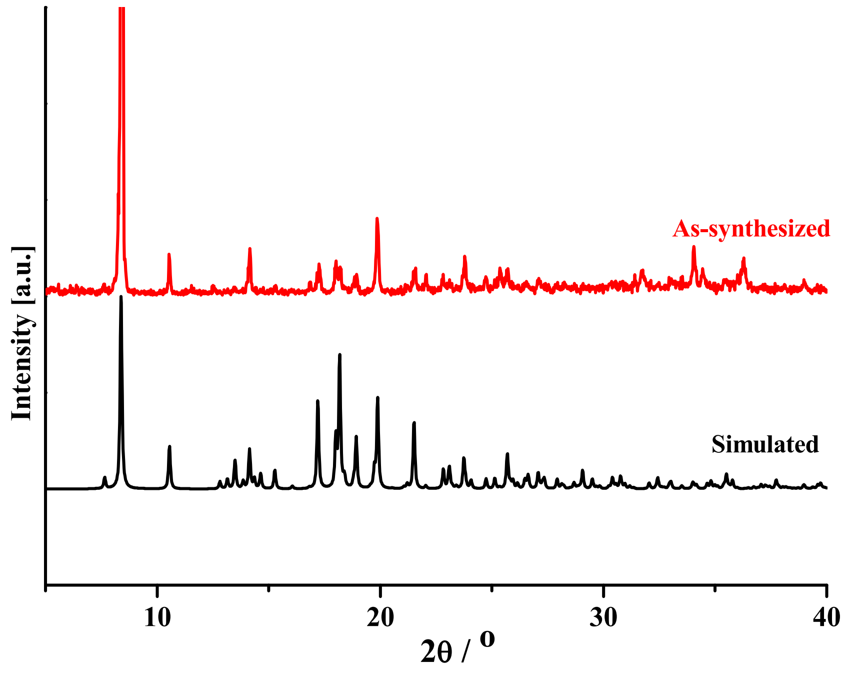

Fig. S11. PXRD pattern of **2**

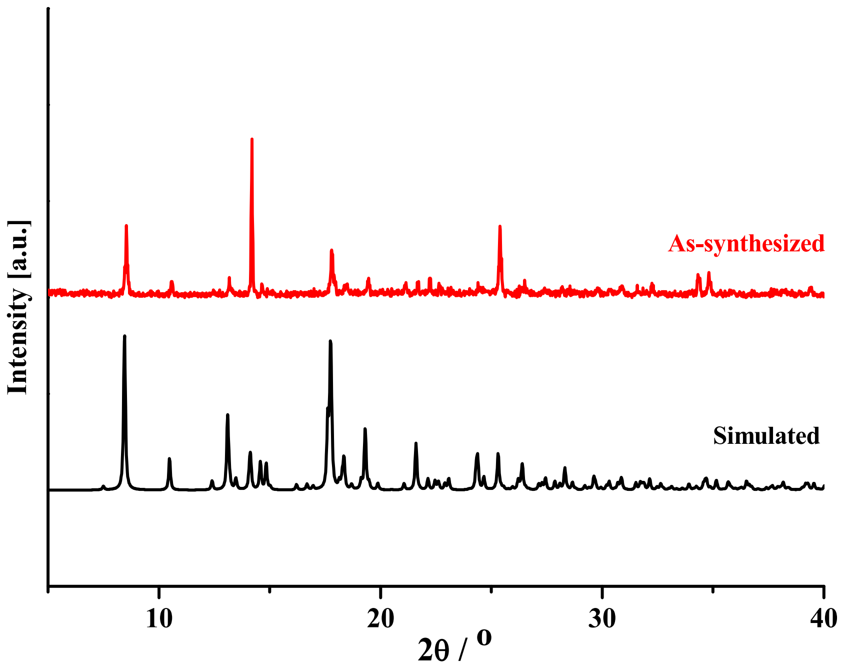

Fig. S12. PXRD pattern of **3**

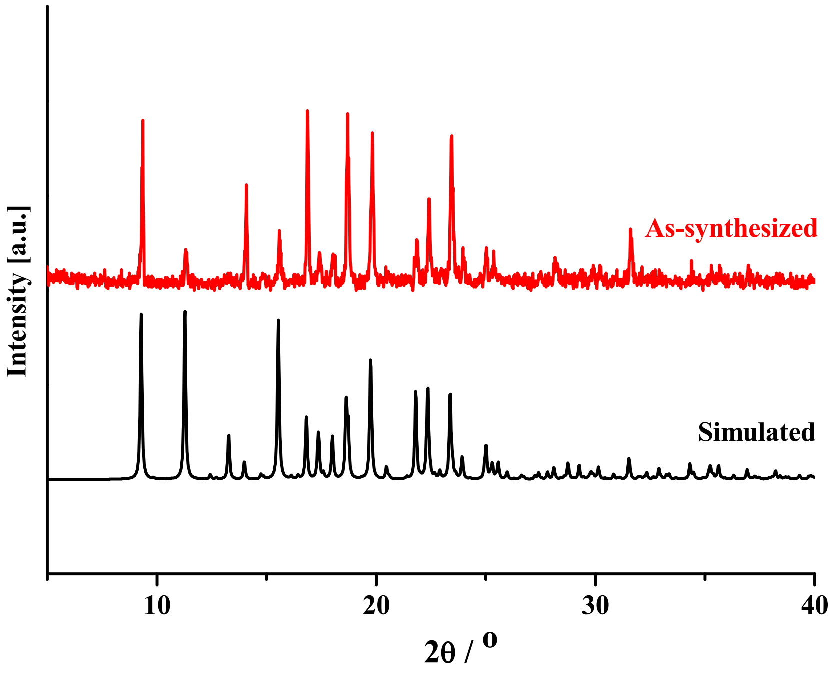

Fig. S13. PXRD pattern of **4**

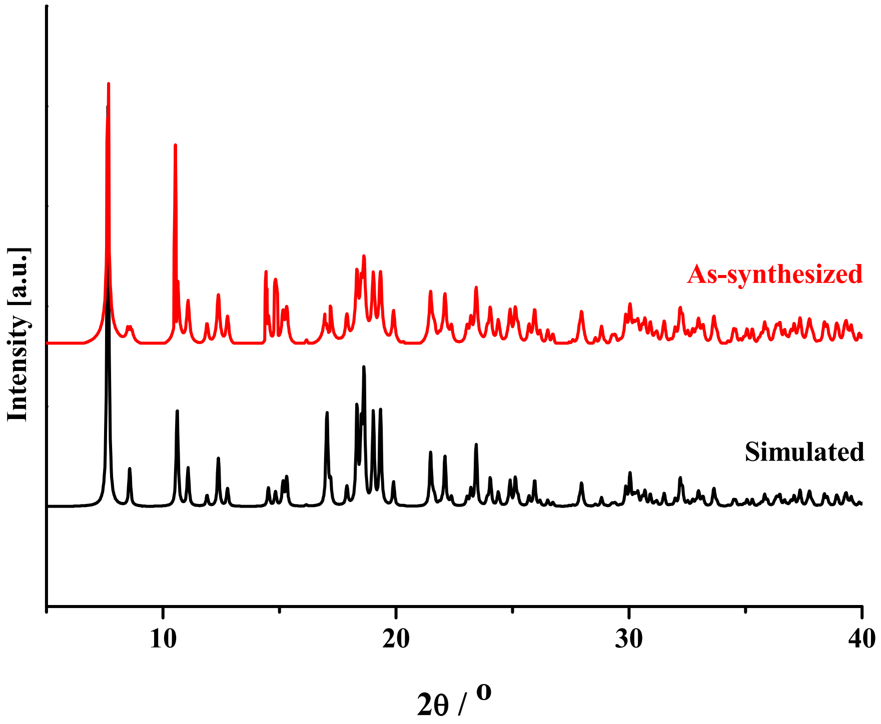

Fig. S14. PXRD pattern of **5**

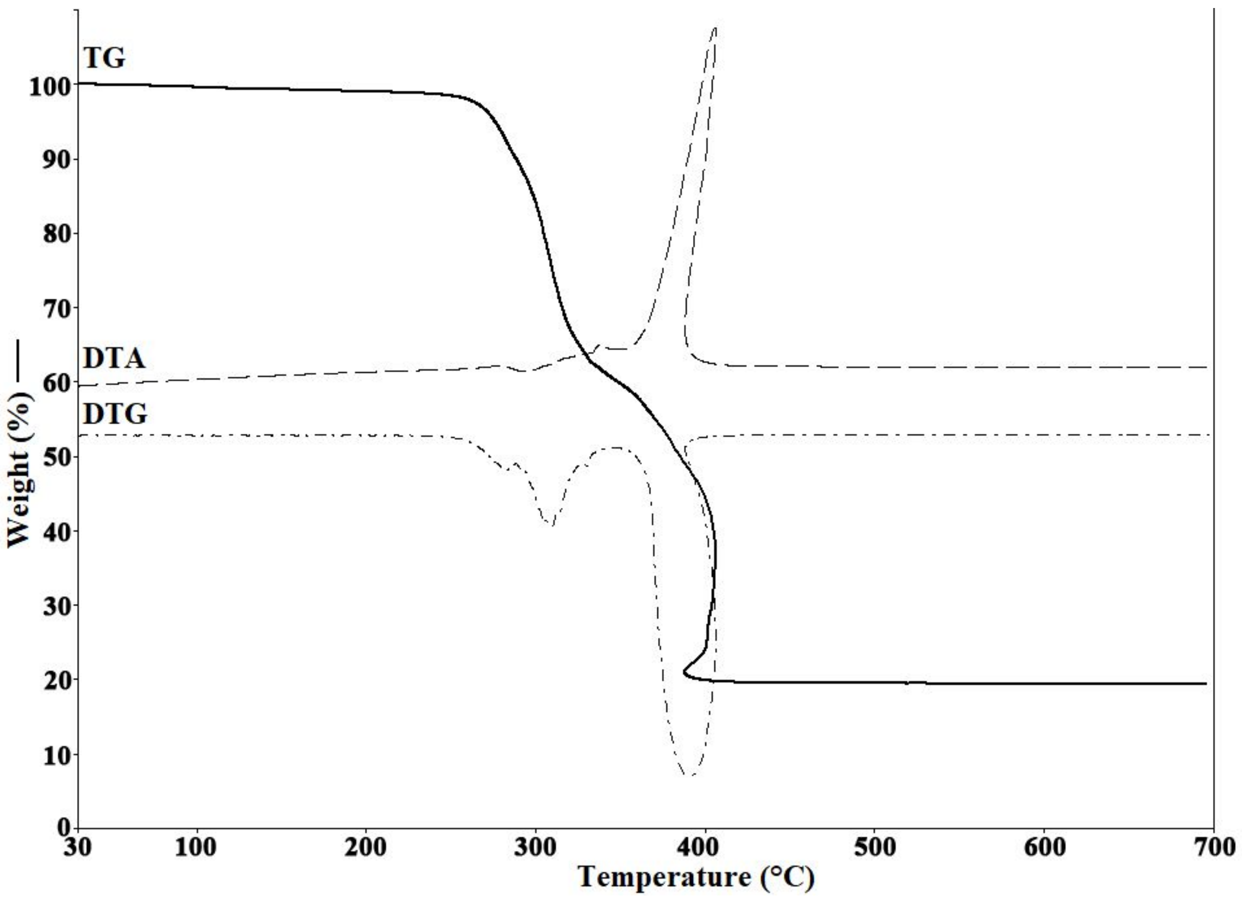

Fig. S15. Thermal analysis curves of **1**

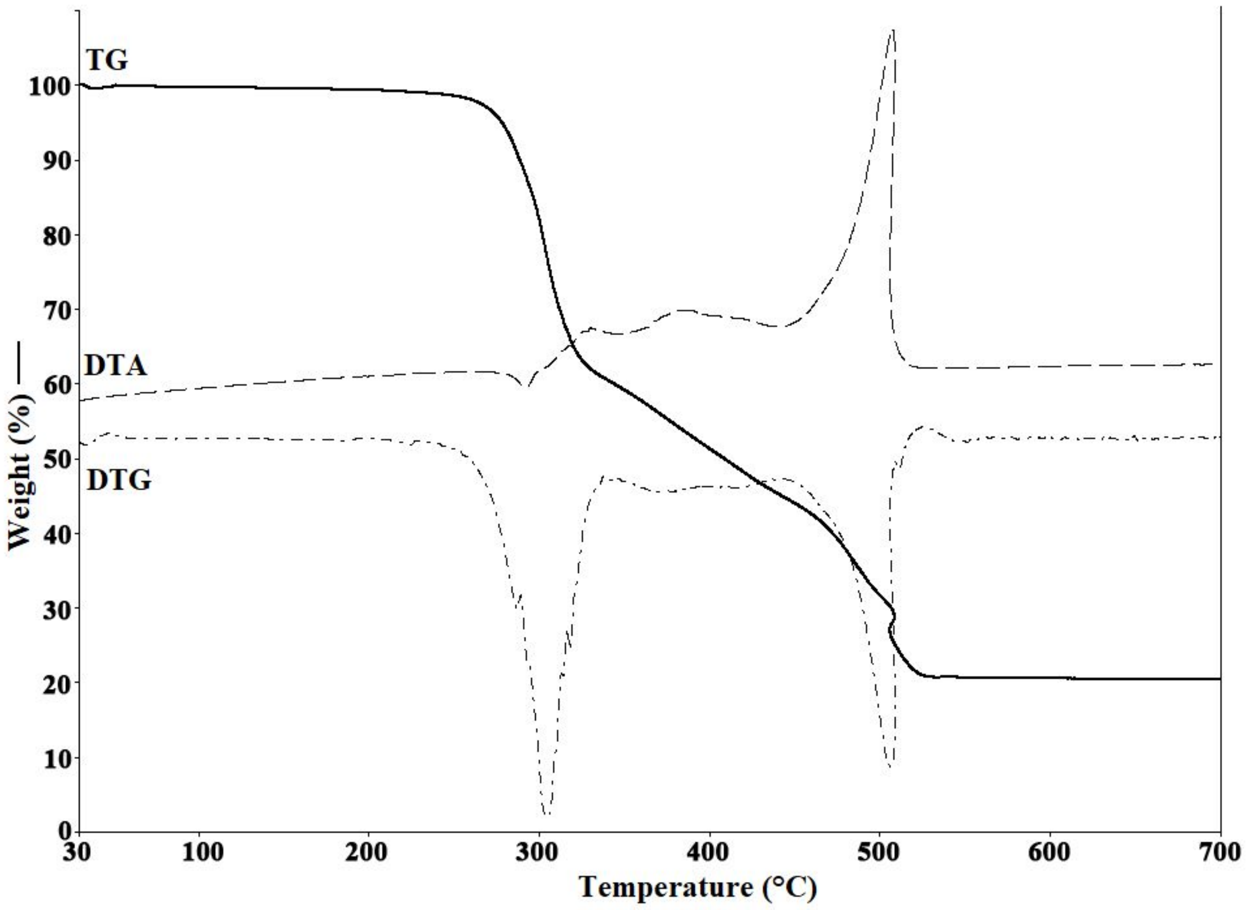

Fig. S16. Thermal analysis curves of **2**

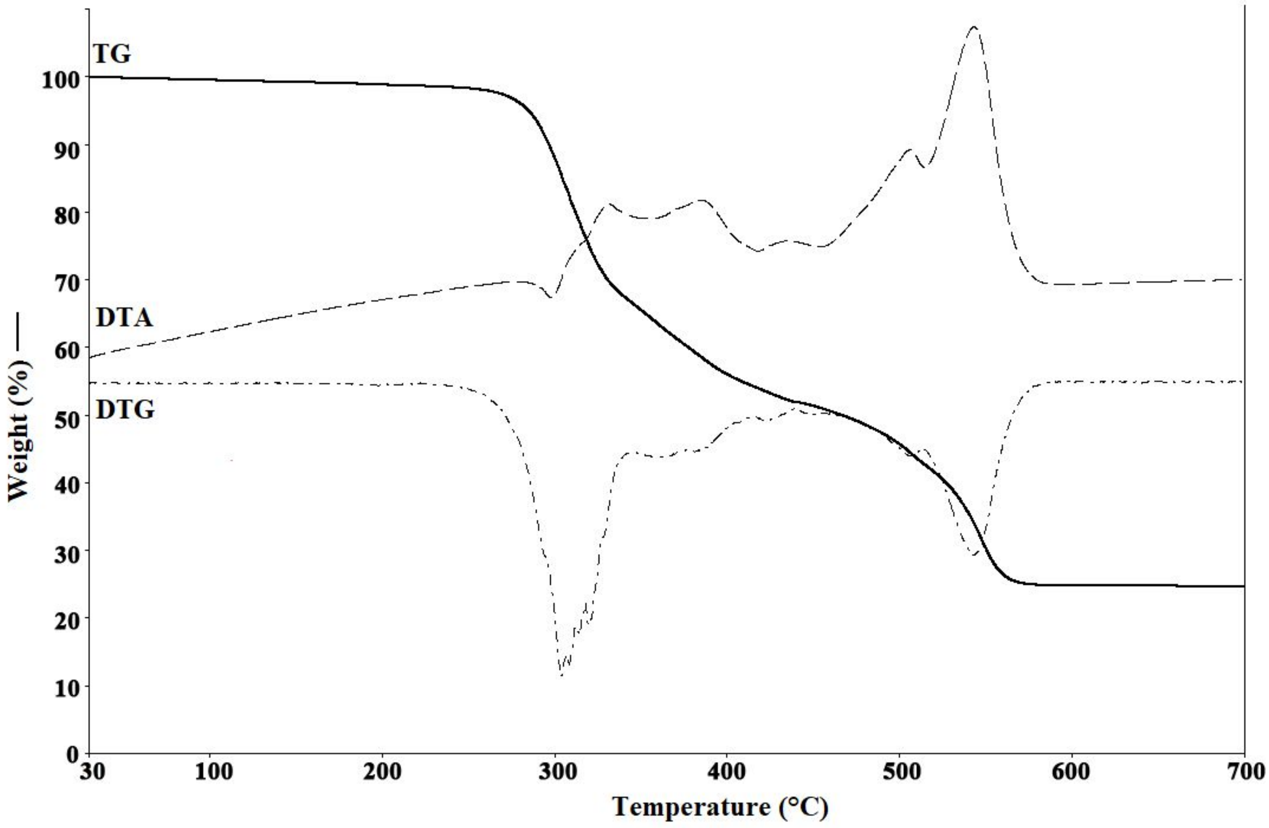

Fig. S17. Thermal analysis curves of **3**

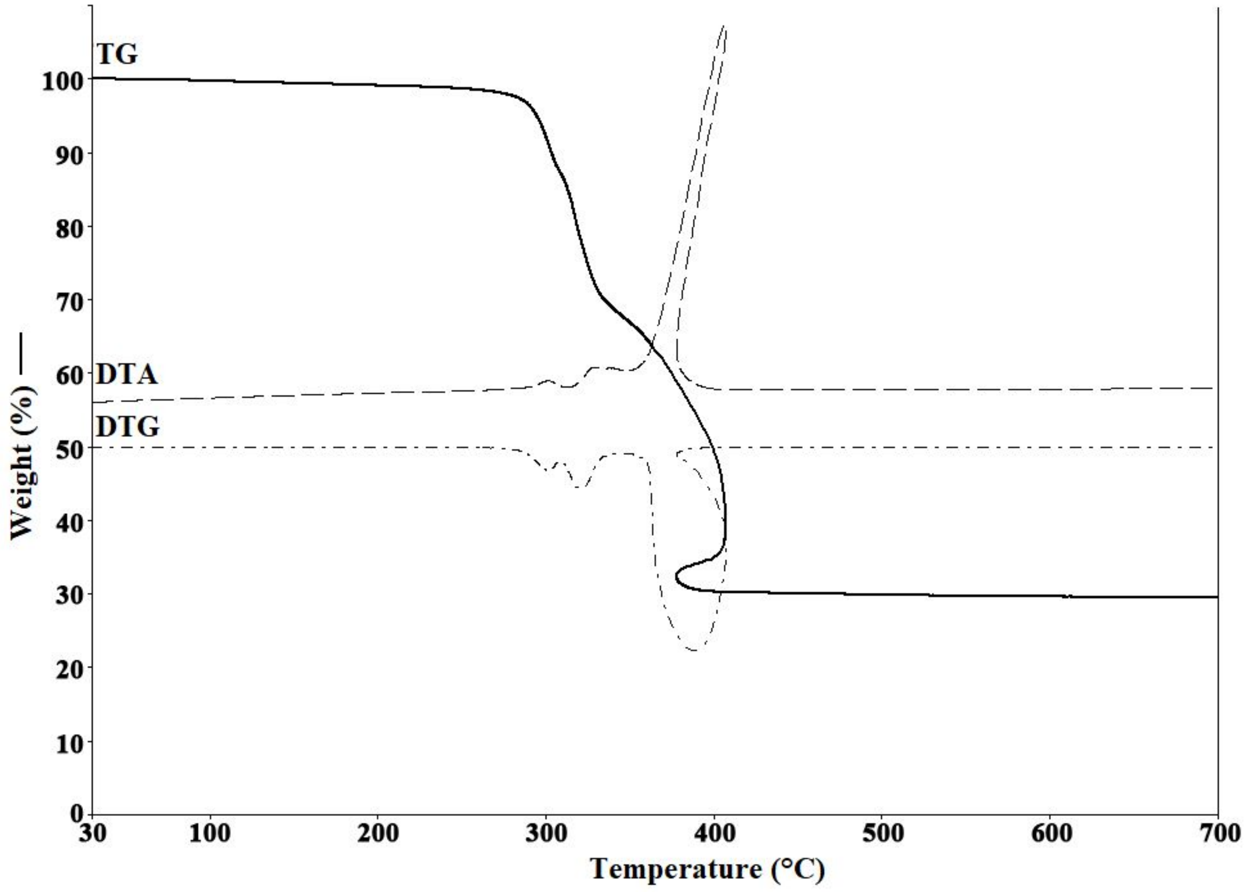

Fig. S18. Thermal analysis curves of **4**

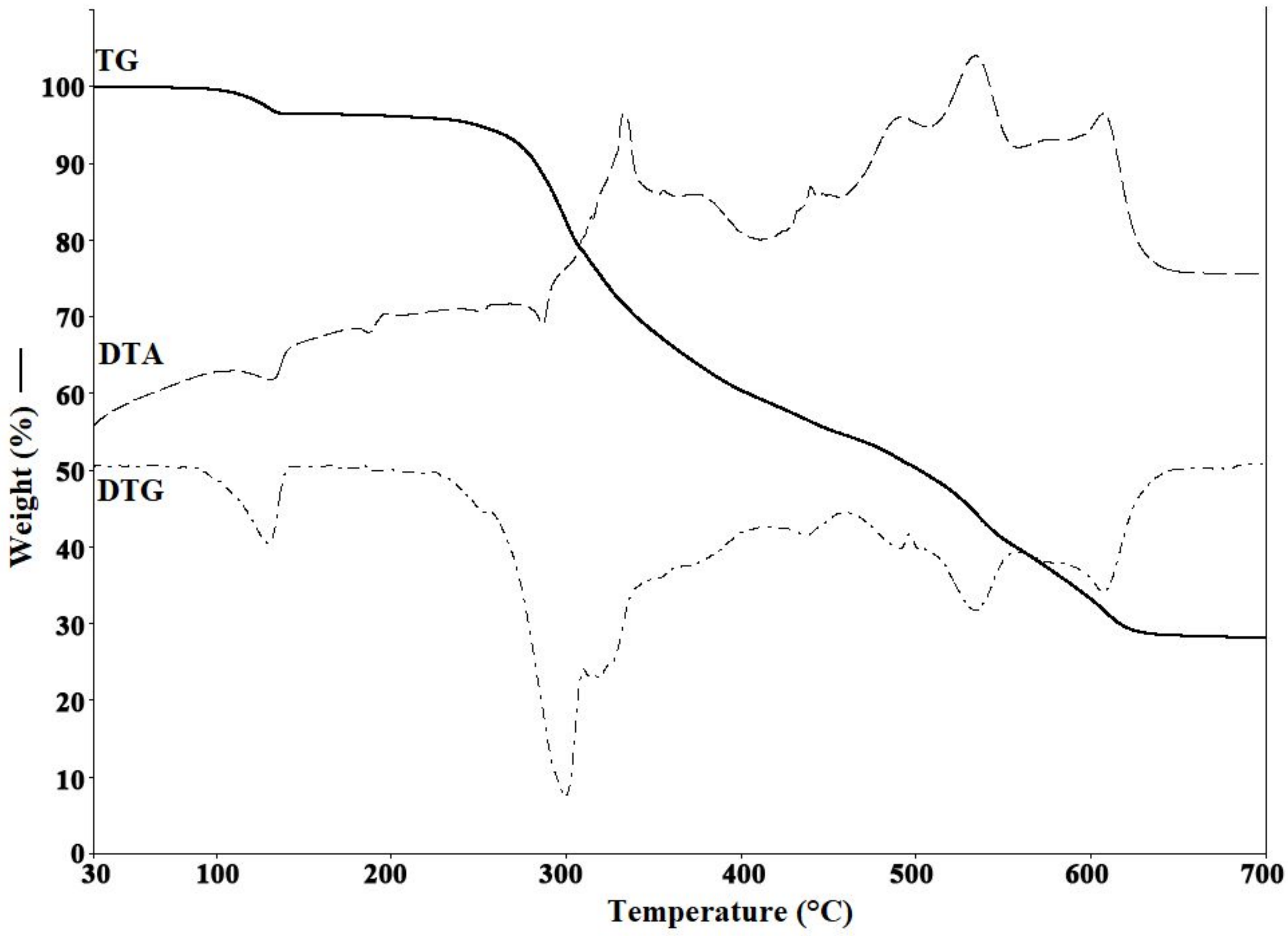

Fig. S19. Thermal analysis curves of **5**

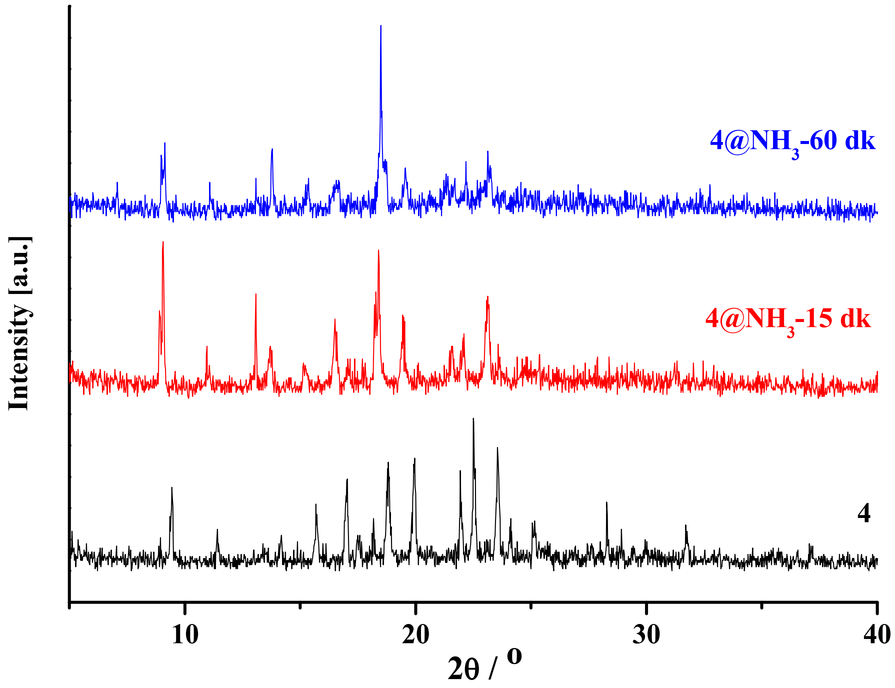

Fig. S20. PXRD pattern of **4** and **4@NH$_3$.**

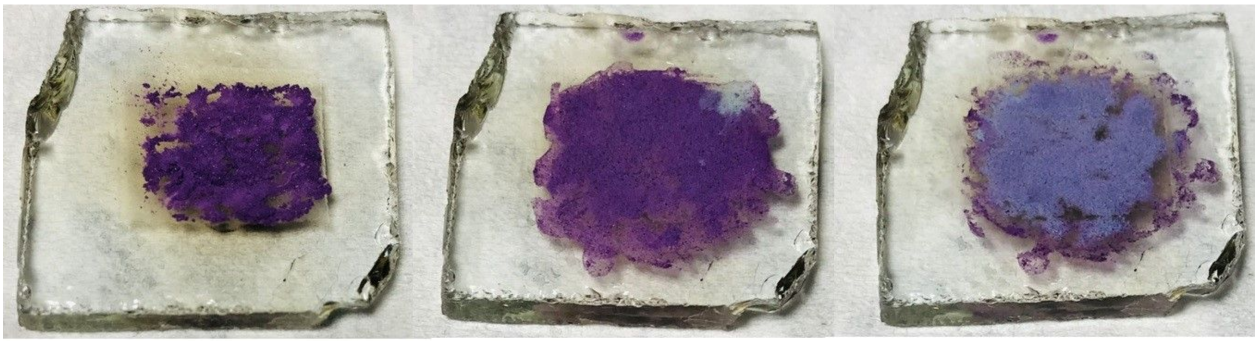

Before NH$_3$ Vapor After 15 minute NH$_3$ Vapor After 60 minute NH$_3$ Vapor

Fig. S21. Photographs of EC polymeric matrix containing CP 4, before and after illumination

Table S1. Selected bond distance (Å) and angle (°) data for **1***

**Bond Lengths (Å)**			
Co1—O4^{i}	2.290 (2)	Co1—N6^{ii}	2.060 (2)
Co1—O1	1.989 (2)	Co1—N1	2.087 (2)
Co1—O3^{i}	2.033 (2)		
**Angles (º)**			
O1—Co1—O4^{i}	161.23 (9)	O3^{i}—Co1—N6^{ii}	121.81 (10)
O1—Co1—O3^{i}	105.11 (10)	O3^{i}—Co1—N1	122.29 (11)
O1—Co1—N6^{ii}	113.22 (10)	N6^{ii}—Co1—O4^{i}	85.38 (8)
O1—Co1—N1	89.94 (10)	N6^{ii}—Co1—N1	100.31 (9)
O3^{i}—Co1—O4^{i}	60.26 (8)	N1—Co1—O4^{i}	89.06 (9)

***Symmetry codes:** (i) −x+1/2, y+1/2, −z+3/2; (ii) −x+5/2, y−1/2, −z+3/2; (iii) −x+1/2, y−1/2, −z+3/2; (iv) −x+5/2, y+1/2, −z+3/2.

Table S2. Selected bond distance (Å) and angle (°) data for **2***

**Bond Lengths (Å)**			
Zn1—O1	1.981 (3)	Zn1—O4^{ii}	2.371 (4)
Zn1—N1	2.042 (3)	Zn1—O3^{ii}	2.092 (4)
Zn1—N6^{i}	2.084 (3)		
**Angles (º)**			
O1—Zn1—N1	116.41 (15)	N1—Zn1—O4^{ii}	86.84 (13)
O1—Zn1—N6^{i}	90.77 (14)	N1—Zn1—O3^{ii}	120.40 (16)
O1—Zn1—O4^{ii}	156.27 (15)	N6^{i}—Zn1—O4^{ii}	89.27 (14)
O1—Zn1—O3^{ii}	102.54 (17)	N6^{i}—Zn1—O3^{ii}	122.89 (17)
N1—Zn1—N6^{i}	100.76 (14)	O3^{ii}—Zn1—O4^{ii}	58.39 (16)

***Symmetry codes:** (i) −x+3/2, y+1/2, −z+1/2; (ii) −x−1/2, y+1/2, −z+1/2; (iii) −x+3/2, y−1/2, −z+1/2; (iv) −x−1/2, y−1/2, −z+1/2.

Table S3. Selected bond distance (Å) and angle (°) for **3***

**Bond Lengths (Å)**			
Cd1—O3^{i}	2.328 (2)	Cd1—N6^{ii}	2.297 (2)
Cd1—O4^{i}	2.360 (2)	Cd1—O1	2.337 (2)
Cd1—N1	2.289 (2)	Cd1—O2	2.321 (2)
**Angles (º)**			
O3^{i}—Cd1—O4^{i}	55.33 (7)	N6^{ii}—Cd1—O4^{i}	125.57 (8)
O3^{i}—Cd1—O1	137.06 (8)	N6^{ii}—Cd1—O1	91.59 (8)
N1—Cd1—O3^{i}	125.79 (8)	N6^{ii}—Cd1—O2	122.18 (10)
N1—Cd1—O4^{i}	85.06 (7)	O1—Cd1—O4^{i}	142.55 (8)
N1—Cd1—N6^{ii}	95.20 (8)	O2—Cd1—O3^{i}	91.78 (9)
N1—Cd1—O1	97.09 (8)	O2—Cd1—O4^{i}	95.25 (9)
N1—Cd1—O2	131.01 (10)	O2—Cd1—O1	55.59 (8)
N6^{ii}—Cd1—O3^{i}	83.20 (7)		

* **Symmetry codes:**(i) −x−1/2, y−1/2, −z+3/2; (ii) −x+3/2, y−1/2, −z+3/2; (iii)

Table S4. Selected bond distance (Å) and angle (°) for **4***

**Bond Lengths (Å)**			
Co1—O1	2.377 (4)	Co2—O7^{iii}	2.318 (5)
Co1—O2	2.032 (4)	Co2—N7	2.051 (5)
Co1—O4^{i}	2.075 (4)	Co2—N6	2.075 (5)
Co1—N12^{ii}	2.078 (4)	Co2—O5	1.996 (4)
Co1—O3^{i}	2.253 (4)	Co2—O8^{iii}	2.027 (4)
Co1—N1	2.081 (5)		
**Angles (º)**			
O2—Co1—O1	58.86 (13)	N1—Co1—O1	85.38
O2—Co1—O4^{i}	105.34 (16)	N1—Co1—O3^{i}	86.99
O2—Co1—N12^{ii}	91.30 (15)	N7—Co2—O7^{iii}	84.91
O2—Co1—O3^{i}	163.74 (17)	N7—Co2—N6	108.1 (2)
O2—Co1—N1	109.04 (17)	N6—Co2—O7^{iii}	85.95
O4^{i}—Co1—O1	86.16 (15)	O5—Co2—O7^{iii}	161.7 (2)
O4^{i}—Co1—N12^{ii}	103.02 (17)	O5—Co2—N7	113.4 (2)
O4^{i}—Co1—O3^{i}	59.75 (18)	O5—Co2—N6	89.0 (2)
O4^{i}—Co1—N1	133.21 (18)	O5—Co2—O8^{iii}	106.5 (2)
N12^{ii}—Co1—O1	150.15 (15)	O8^{iii}—Co2—O7^{iii}	59.00
N12^{ii}—Co1—O3^{i}	86.40 (18)	O8^{iii}—Co2—N7	124.9 (2)
N12^{ii}—Co1—N1	106.87 (19)	O8^{iii}—Co2—N6	109.0 (2)
O3^{i}—Co1—O1	122.00 (16)		

* **Symmetry codes:** (i) x−1/2, −y, z; (ii) x, y−1, z; (iii) x+1/2, −y+1, z;

Table S5. Selected bond distance (Å) and angle (°) for **5***

**Bond Lengths (Å)**			
Cd1–O4^{i}	2.5473 (17)	Cd1–O5	2.2670
Cd1–O3^{i}	2.3662 (16)	Cd1–N4	2.3287
Cd1–O1	2.2710 (17)	Cd1–N1	2.320 (2)
**Angles (º)**			
O3^{i}–Cd1–O4^{i}	52.89 (5)	O5–Cd1–N4	86.89 (7)
O1–Cd1–O4^{i}	140.15 (6)	O5–Cd1–N1	89.27 (8)
O1–Cd1–O3^{i}	87.87 (6)	N4–Cd1–O4^{i}	80.31 (6)
O1–Cd1–N4	106.91 (6)	N4–Cd1–O3^{i}	86.58 (7)
O1–Cd1–N1	90.93 (7)	N1–Cd1–O4^{i}	81.59 (7)
O5–Cd1–O4^{i}	96.83 (7)	N1–Cd1–O3^{i}	87.36 (7)
O5–Cd1–O3^{i}	149.70 (7)	N1–Cd1–N4	160.91
O5–Cd1–O1	122.29 (7)		

* **Symmetry codes:** (i) x+1, y, z; (ii) x−1, y, z; (iii) −x+1, −y+1, −z+2; (iv) −x+2, [illegible]
